# Intrinsic functional clustering of the macaque insular cortex

**DOI:** 10.3389/fnint.2023.1272529

**Published:** 2024-01-05

**Authors:** Lotte Sypré, Saloni Sharma, Dante Mantini, Koen Nelissen

**Affiliations:** ^1^Laboratory for Neuro- & Psychophysiology, Department of Neurosciences, KU Leuven, Leuven, Belgium; ^2^Leuven Brain Institute, KU Leuven, Leuven, Belgium; ^3^Harvard Medical School, Boston, MA, United States; ^4^Movement Control & Neuroplasticity Research Group, KU Leuven, Leuven, Belgium

**Keywords:** macaque, insula, clustering, resting-state, fMRI

## Abstract

The functional organization of the primate insula has been studied using a variety of techniques focussing on regional differences in either architecture, connectivity, or function. These complementary methods offered insights into the complex organization of the insula and proposed distinct parcellation schemes at varying levels of detail and complexity. The advent of imaging techniques that allow non-invasive assessment of structural and functional connectivity, has popularized data-driven connectivity-based parcellation methods to investigate the organization of the human insula. Yet, it remains unclear if the subdivisions derived from these data-driven clustering methods reflect meaningful descriptions of the functional specialization of the insula. In this study, we employed hierarchical clustering to examine the cluster parcellations of the macaque insula. As our aim was exploratory, we examined parcellations consisting of two up to ten clusters. Three different cluster validation methods (fingerprinting, silhouette, elbow) converged on a four-cluster solution as the most optimal representation of our data. Examining functional response properties of these clusters, in addition to their brain-wide functional connectivity suggested a functional specialization related to processing gustatory, somato-motor, vestibular and social visual cues. However, a more detailed functional differentiation aligning with previous functional investigations of insula subfields became evident at higher cluster numbers beyond the proposed optimal four clusters. Overall, our findings demonstrate that resting-state-based hierarchical clustering can provide a meaningful description of the insula’s functional organization at some level of detail. Nonetheless, cluster parcellations derived from this method are best combined with data obtained through other modalities, to provide a more comprehensive and detailed account of the insula’s complex functional organization.

## 1 Introduction

In the last few decades, there has been a renewed interest in studying the functional organization of the insula, due to accumulating evidence suggesting its role in a variety of important affective, cognitive and regulatory functions (Craig, 2009; Nieuwenhuys, 2012; Evrard, 2019) and in a host of mental disorders and dysfunctions (Nagai et al., 2007; Nomi et al., 2019). Differentiation within the primate insula has previously been examined using several criteria including structure (cyto-, myelo- and receptor architecture), function (single cell properties, fMRI responses or behavioral effects after perturbations/lesions) and connectivity (structural or functional) between neighbouring insular subdivisions. Data obtained through the aforementioned techniques have provided information at different spatial scales about the functional specialization and integration of information processing in the insula. This work has led to the proposal of several coarse or more fine-grained parcellation schemes for the human and non-human primate insulae.

For instance, earlier architectonic studies proposed a tripartite organization dividing human and monkey insula into a posterior granular, middle dysgranular and anterior agranular sector (Mesulam and Mufson, 1982, 1985). However, other examinations suggested a more complex arrangement of insular architectonic sectors, further dividing the granular, dysgranular and agranular sectors into a larger number of distinct sub-regions (Brockhaus, 1940; Kurth et al., 2010a,b; Gallay et al., 2012; Morel et al., 2013; Evrard et al., 2014; Quabs et al., 2022). Moreover, numerous electrophysiology and task-based fMRI studies have provided additional evidence for the functional specialization of insular subregions, proposing specific subsectors that process a wide variety of intero- and/or exteroceptive signals (Yaxley et al., 1990; Schneider et al., 1993; Verhagen et al., 2004; Remedios et al., 2009; Chen et al., 2010; Caruana et al., 2011; Jezzini et al., 2012; Avery et al., 2015; Sharma et al., 2018; Mazzola et al., 2019; Soyman et al., 2022; Yang et al., 2022; Sypré et al., 2023). Connectivity measurements, mainly involving invasive tract tracing experiments in macaques, have provided further support for the proposed functional specializations of the insula (Mesulam and Mufson, 1982; Friedman et al., 1986; Gerbella et al., 2011; Jezzini et al., 2015; for detailed review see Evrard, 2019).

Recently, non-invasive neuroimaging techniques that allow mapping structural connectivity using diffusion-weighted MRI or functional connectivity using resting-state fMRI have become popular tools to characterize the organization of the human and non-human primate insula (Taylor et al., 2009; Cauda et al., 2011; Cerliani et al., 2012; Cloutman et al., 2012; Jakab et al., 2012; Kelly et al., 2012; Touroutoglou et al., 2016; Ghaziri et al., 2017; Nomi et al., 2018; Di Cesare et al., 2019; Klugah-Brown et al., 2022; Sypré et al., 2023). While several resting-state studies used *a priori* defined subregions or seeds (Taylor et al., 2009; Cauda et al., 2011; Ebisch et al., 2011; Touroutoglou et al., 2012, 2016; Sypré et al., 2023), another popular approach involves using a data-driven clustering method. This technique parcellates the insula into distinct subregions based on the similarity of the connectivity profiles of the individual insular voxels. Due to a wide range of possible experimental and methodological parameters when performing data-driven clustering analyses (for extensive review, see Eickhoff et al., 2015), it is not surprising that parcellation schemes of the human insula derived from data-driven clustering techniques vary across studies. For instance, while some studies propose a bipartite or tripartite (Cauda et al., 2011, 2012; Deen et al., 2011; Chang et al., 2013; Ryali et al., 2015) subdivision for the insula, others suggest a higher number of subdivisions (Kelly et al., 2012; Ryali et al., 2013; Glasser et al., 2016).

In macaques, it is unclear whether data-driven clustering of resting-state signals could retrieve an organization of the insula that aligns with the partitioning schemes suggested by invasive techniques. In this study, we employed data-driven hierarchical clustering using intrinsic resting-state functional connectivity (Hutchison and Everling, 2014; Schaeffer et al., 2019a,b, 2020; Sharma et al., 2021) to examine resting-state clustering-derived subdivisions of the macaque insula. To functionally differentiate these clusters, we examined their response profile using a range of task-based fMRI data sets that were recently used to investigate the functional specialization of the macaque insula (Sypré et al., 2023). Finally, we also employed seed-based analyses to examine if the clusters differed in terms of their functional connectivity with the rest of the brain.

## 2 Materials and methods

### 2.1 Subjects

Eight rhesus monkeys (*Macaca mulatta*, M1 – M8, 6 males, 2 females, 5–8 kg) were scanned for the awake resting-state fMRI experiments. To functionally characterize the intrinsic functional connectivity derived insular clusters, we used these clusters as regions-of-interest (ROIs) on a range of task-based fMRI datasets from Sypré et al. (2023). Most of these task-based fMRI datasets were acquired in two of the monkeys (M1 and M2, both males) also included in the resting-state group. The galvanic vestibular stimulation experiment (see below) included two additional monkeys (M9 and M10, *Macaca mulatta*, both females, 5–8 kg). Animal care and experimental procedures followed the national and European guidelines and were approved by either the animal ethical committee of KU Leuven (experiments involving monkeys M1 – M8) or the French Ministry of Research (MP/03/34/10/09) and the local ethics committee of Toulouse University (CNREEA code: C2EA–14) (experiments involving monkeys M9 and M10).

### 2.2 Awake resting-state fMRI

During training and scanning sessions, animals sat in a sphinx position in a custom-made MR-compatible chair while they had to fixate within a 2 × 2° window centered on a red dot (0.35 × 0.35°) in the center of a screen positioned in front of them. The subjects received juice rewards continuously throughout the scans for maintaining fixation while eye position was monitored (at 120 Hz) through pupil position and corneal reflection (Iscan). The interval between rewards was systematically decreased (from 2,500 to 800 ms) as the monkeys maintained their fixation within the fixation window. These scans are considered ‘resting-state’ in a sense that there were no specific changes in cognitive or behavioral demands during different parts of the scans, as is typical for task-based fMRI. That said, it is possible that overall strength of functional coupling between regions differs depending on the exact behavior of the subject (fixation vs. no fixation, eyes open vs. eyes closed, reward vs. no reward, etc.) but this has not been tested directly in monkeys (due to the fact that scanning awake monkeys without using rewards can result in excessive body movements of the animal leading to poor data quality). Similar fixation tasks were previously employed both in monkey (Mantini et al., 2011; Touroutoglou et al., 2016; Sharma et al., 2019, 2021; Giacometti et al., 2022) and human resting-state studies (Patriat et al., 2013; Spadone et al., 2015; Agcaoglu et al., 2019). Resting-state scans involving fixation tasks have been used by several groups to avoid alterations between sleep and wakefulness (Tagliazucchi and Laufs, 2014) and in combination with juice rewards avoid excessive movements when acquiring fMRI data in awake monkeys (Vanduffel et al., 2001). One run lasted 10 min and consisted of 300 volumes. For the macaque resting-state fMRI analysis, runs with a fixation percentage below 85% were excluded, which encompassed a minority of all data acquired. In addition, the average correlation across voxel time-series was calculated for each run. Runs with values below or above two times the median across runs were considered as outliers and excluded (number of excluded runs per subject: M1: 0 runs, M2: 1 run, M3: 2 runs, M4: 1 run, M5: 2 runs, M6: 1 run, M7: 1 run, M8: 1 run) from data analysis (Mantini et al., 2011; Sharma et al., 2019). This resulted in 18 runs from monkey M1, 19 runs from monkey M2, 17 runs from monkey M3, 19 runs from monkey M4, 18 runs from monkey M5, 14 runs from monkey M6, 15 runs from monkey M7, and 19 runs from monkey M8. Resting-state fMRI data used in this study are the same data as also used in previously published studies by our lab (Sharma et al., 2019, 2021; Sypré et al., 2023).

### 2.3 Task-based fMRI experiments

Functional response properties of intrinsic connectivity-derived clusters were examined using a range of functional MRI task localizers used recently to examine functional MRI responses to different sensory stimulations in the macaque insula (Sypré et al., 2023). These task localizers examined taste responses, distaste/disgust responses, somato-motor responses related to grasping movements under haptic feedback, vestibular responses and visual responses during observation of conspecifics’ emotional facial expressions. The task localizer data used in this study are the same as those used and described in detail in Sypré et al. (2023).

#### 2.3.1 Taste localizer

During the taste coding experiment, monkeys had to fixate within a 2 × 2° window centered around a red fixation point (0.35 × 0.35°) positioned in the center of the screen while eye movements were recorded (Iscan). During fixation, monkeys received random blocks of different liquid tastants: sweet (0.3 M sucrose), sour (0.01 M citric acid) or distilled water (baseline). Sweet and sour taste solutions were prepared using distilled water. Each block consisted of 10 volumes (20 s) and was followed by 5 volumes (10 s) of tasteless baseline (distilled water) to eliminate any residual taste. This sequence was repeated thrice per run, resulting in 185 volumes (6 min 10 s). Conditions were presented randomly across runs.

#### 2.3.2 Distaste/disgust localizer

For the distaste/disgust fMRI localizer, monkeys were required to maintain fixation within a 2 × 2° window centered around a red fixation point (0.35 × 0.35°) positioned in the center of the screen while eye movements were recorded (Iscan). While fixating, blocks of two different concentrations of sour solution (0.01 M or 1 M citric acid, dissolved in distilled water) were presented in a random order across runs. Distilled water was used as baseline. One run lasted for 240 volumes (8 min), where alternating blocks of high sour, low sour and distilled water lasted for 10 s and were followed by 20 s of distilled water to rinse out residual taste. The sequence of conditions was repeated four times per run.

#### 2.3.3 Grasping execution localizer

The grasping motor task has been previously described in detail in Sharma et al. (2018) and Sypré et al. (2023). A block design was used and monkeys performed two motor tasks in the dark: reach-and-grasp execution and reach-only execution. In addition, a fixation-only condition was included as baseline. For the reach-and-grasp execution task, monkeys had to grasp three spheres of different sizes (16, 23 or 40 mm radius, respectively) with their right hand. Monkeys also performed a reach-only motor task as control for the reach-and-grasp task. During the fixation baseline condition, the monkeys needed to maintain fixation within a 2 × 2° window centered around a red fixation point (0.35 × 0.35°) positioned in the center of the screen while keeping their right hand in the start position in order to receive a liquid reward (Nelissen and Vanduffel, 2011; Nelissen et al., 2018; Sharma et al., 2018). Eye movements were recorded during the experiments (Iscan). Functional MRI runs of the grasping localizer experiment consisted of alternating blocks of reach-and-grasp, reach-only and fixation-only trials (Sharma et al., 2018). A typical run consisted of five start volumes followed by a sequence of blocks of grasp 16 mm sphere – fix only – grasp 40 mm sphere – fix only – grasp 23 mm sphere – fix only – reach-only – fix only – fixation only (baseline) – fix only. Each of these blocks lasted for 30 s and this sequence was repeated once per run, which resulted in 305 volumes (10 min 10 s) per run.

#### 2.3.4 Visual facial expression localizer

During the facial expression localizer, several visual stimuli were presented while monkeys were required to fixate within a 2 × 2° window centered on a red dot (0.35 × 0.35°) in the center of the screen. Animals received juice rewards for maintaining fixation. Eye position was monitored through pupil position and corneal reflection (Iscan). Visual stimuli included second-person perspective video clips of conspecific emotional lip-smacking facial expressions. In addition scrambled control stimuli for each lip-smack video were made by phase scrambling each frame of the corresponding video sequence using MATLAB (Nelissen et al., 2005, 2006). In total, stimulus set included eight different lip-smack videos and their corresponding scrambled controls. All videos measured 11.8 × 11.8 visual degrees and had lasted 2 s. A typical run of the facial expression localizer lasted 185 volumes (6 min 10 s) and consisted of five start volumes followed by a sequence of blocks of lip-smacking (30 s) – fixation only (10 s) – scrambled (30 s) – fixation only (10 s) – fixation only (30 s) – fixation only (10 s). This sequence of blocks (totalling 120 s or 60 volumes) was presented consecutively three times per run. Order of conditions were randomized across runs. Within each lip-smacking or scrambled block, 15 videos were randomly presented out of the stimuli batch of eight videos.

#### 2.3.5 Galvanic vestibular localizer

Galvanic vestibular stimulation was performed by two gold electrodes (diameter 1 cm), placed on the mastoid behind the ears of the two subjects (M9 and M10). Stimulation consisted of a sinusoidal current with a frequency of 1 Hz and an amplitude of ±2.5 mA. To regulate the frequency and amplitude of the voltage, a computer in the MRI control room sent a voltage to a USB data acquisition card (NI USB-6009, DAQ 8 AD 2DA 14 bit 48kS/s Labview). The NI card redirected the voltage to a current-limited stimulator (Digitimer, UK; model DS5) which transformed this voltage into current and, after passing through the MR filter, delivered the resulting current into the electrodes placed behind the ears of the monkey. During the galvanic vestibular stimulation experiments, monkey subjects were anesthetized using a mixture of medetomidine (0.04 mg/kg) and ketamine (10 mg/kg). For the galvanic vestibular stimulation experiment, a block design was used that consisted of blocks of bilateral stimulation or no stimulation. Each block lasted for 12 volumes or 18 s and was repeated 6 times per run (runs included 145 volumes and lasted 3 min 38 s).

### 2.4 fMRI data acquisition

Resting-state and task-based fMRI data involving awake subjects were acquired with a 3.0 Tesla full-body scanner (Siemens) using a gradient-echo T2*-weighted echo-planar imaging sequence of 40 horizontal slices (time repetition (TR) = 2 s, time echo (TE) = 17 ms, flip angle = 90°, 1.25 mm^3^ isotropic voxels). During these experiments, functional images were acquired using a custom-built eight-channel phased-array receive coil and a saddle-shaped, radial transmit-only surface coil (Kolster et al., 2009). Before every scanning session, an iron contrast agent (Molday ION, BioPAL in monkey M1 – M4 and MION or Sinerem, Laboratoir Guerbet in monkey M5 – M8) was injected into the femoral or saphenous vein (6–12 mg/kg) to improve spatial selectivity of MR signal changes, thus improving signal-to-noise ratio (Vanduffel et al., 2001; Zhao et al., 2006). For the galvanic vestibular stimulation experiment in anesthetized animals, a 3.0 Tesla clinical MR scanner (Philips Achieva) was used, in addition to a custom-built eight-channel phased-array coil (RapidBiomed). Blood oxygen level dependent (BOLD) T2*-weighted functional volumes were acquired by gradient echo-planar imaging (GE-EPI: TR = 1,500 ms; TE = 30 ms; flip angle = 75°; SENSE factor = 1.6; voxel size = 1.25 × 1.25 × 1.5 mm^3^; 46 axial slices with no inter-slice gap; FOV = 80 × 80 mm).

### 2.5 fMRI data preprocessing

Data were motion-corrected by spatial realignment of each volume to the first volume of the first run using statistical parametric mapping (SPM12; RRID:SCR_007037). Subsequently, the functional images corrected for motion artefacts were processed with rigid and non-rigid co-registration using JIP software (http://www.nitrc.org/projects/jip/; RRID:SCR_009588) to account for variability across individual monkey’s anatomy. All functional images were co-registered to a template anatomical image (monkey M12; Ekstrom et al., 2008; Nelissen and Vanduffel, 2011). Next, co-registered functional images were resliced to 1 mm^3^ isotropic voxel size and smoothed with SPM12 with a 2.5 mm or 1.5 mm full-width half maximum (FWHM) Gaussian kernel for resting-state fMRI data or task-based fMRI data, respectively (Sypré et al., 2023).

### 2.6 Hierarchical clustering analysis

**Figure 1:**
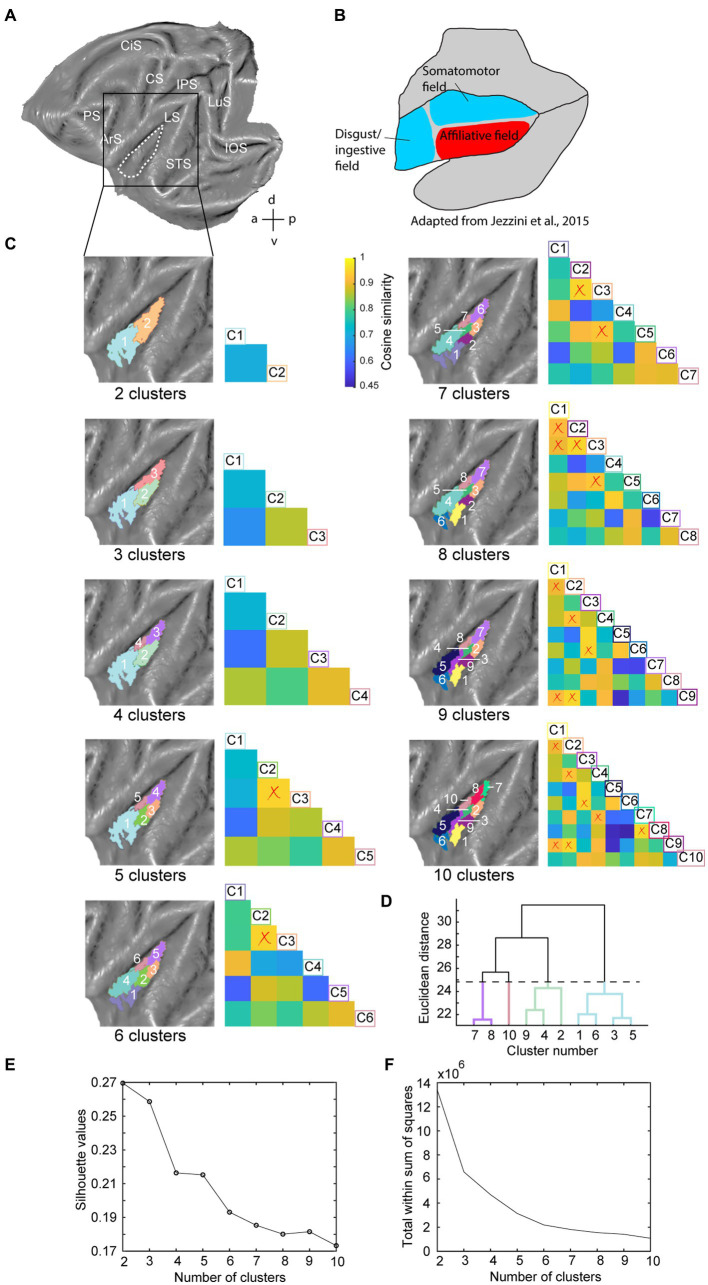
Hierarchical clustering analysis of the insula in the left hemisphere. **(A)** Dashed white line indicates insula mask used in the hierarchical clustering analysis overlaid on a left hemisphere template flatmap. **(B)** Functional organization of the insula as described by Jezzini et al. (2015, adapted). This scheme parcellates the insula in a disgust/ingestive, somato-motor and affiliative field. **(C)** Results of the hierarchical clustering analysis depicting two- until ten-cluster solutions. Within each cluster solution, different colors represent individual clusters. The cosine similarity matrix next to each cluster FIGURE 1 (Continued)solution shows the pairwise comparisons of each cluster with every other cluster in that cluster solution in terms of their extrinsic interareal connectivity (also referred as their fingerprint) with 23 external seeds. The lower limit of the color bar corresponds to the lowest cosine similarity value considering all two- to ten-cluster solution comparisons. Low cosine similarity values indicate that the compared clusters have distinct functional connectivity with the 23 external seeds, i.e., have dissimilar fingerprints. High cosine similarity values on the other hand indicate cluster pairs with a similar functional connectivity with the 23 external seeds (similar fingerprint). Cluster labels in the cosine similarity matrix correspond to clusters in the adjacent flatmap, where the square boxes are color-matched to the clusters. Red crosses overlaid on the similarity matrix indicate pairwise cluster comparisons that do not demonstrate significantly different fingerprints (*p* < 0.05), calculated by a permutation testing approach (see methods). **(D)** Dendrogram of the ten-cluster solution as displayed in the last flatmap of **(C)**. Cluster labels on the x-axis correspond to the cluster numbers displayed on the aforementioned flatmap, where the y-axis displays the Euclidean distance between cluster subdivisions. The four colored branches of the dendrogram indicate which cluster numbers of the ten-cluster solution correspond to the regions of the four-cluster solution, which was selected as optimal cluster solution. **(E)** Silhouette analysis of the two- to ten-cluster solution where the labels on the x-axis correspond to the cluster solutions. **(F)** Elbow analysis of the two to ten-cluster solution. This analysis uses the total within-cluster sum of squares (i.e., a measure of variance within a cluster solution) to assess the optimal number of clusters. LuS – lunate sulcus; IOS – inferior occipital sulcus; STS – superior temporal sulcus; LS – lateral sulcus; IPS – intraparietal sulcus; CS – central sulcus; CiS – cingulate sulcus; ArS – arcuate sulcus; PS – principal sulcus; a: anterior; p: posterior; d: dorsal; v: ventral.

**Figure 2:**
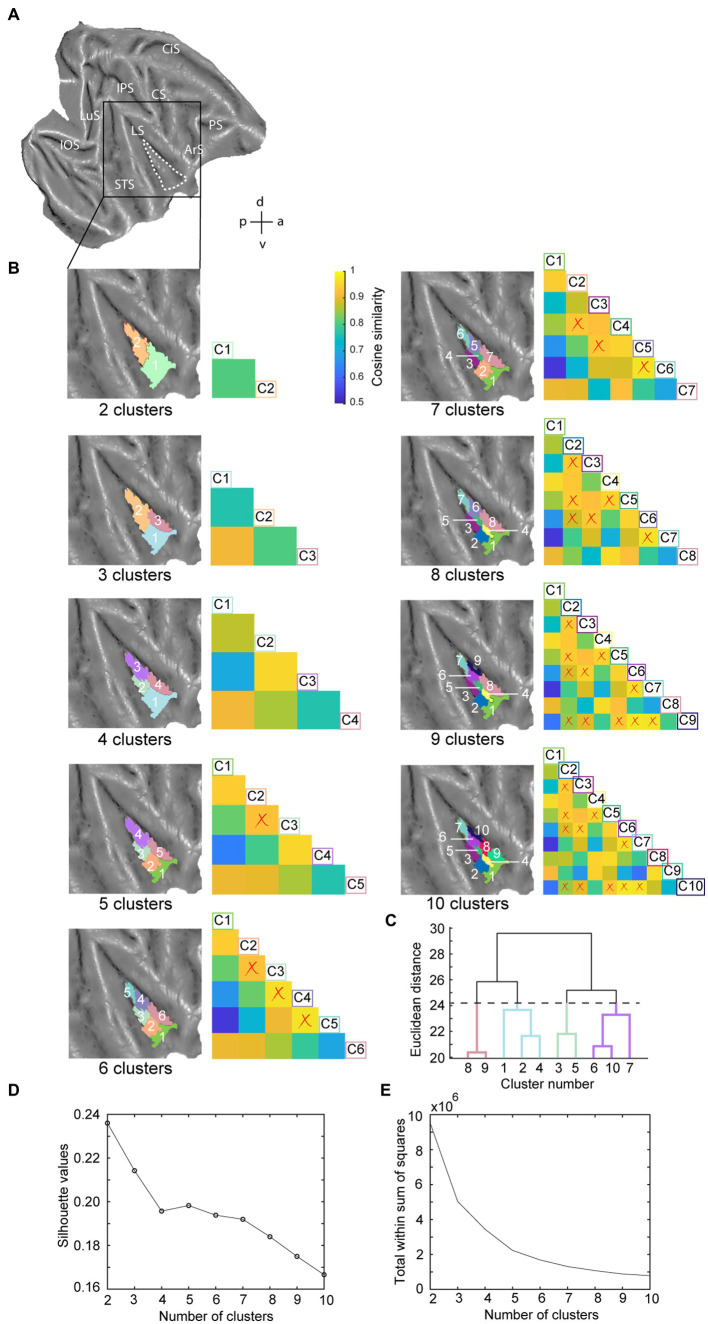
Hierarchical clustering analysis of the insula in the right hemisphere. **(A)** Dashed white line indicates the insula mask used in the hierarchical clustering analysis overlaid on a right hemisphere template flatmap. **(B)** Results of the hierarchical clustering analysis representing the two- until ten-cluster FIGURE 2 (Continued)solutions. Same conventions as Figure 1 are used. **(C)** Dendrogram of the ten-cluster solutions as displayed in the last flatmap of **(B)**. Cluster labels on the x-axis correspond to the cluster numbers displayed on the aforementioned flatmap, where the y-axis displays the Euclidean distance between cluster subdivisions. **(D)** Silhouette analysis of the two- to ten-cluster solutions where the labels on the x-axis correspond to the cluster solutions. **(E)** Elbow analysis of the two to ten cluster solution. LuS – lunate sulcus; IOS – inferior occipital sulcus; STS – superior temporal sulcus; LS – lateral sulcus; IPS – intraparietal sulcus; CS – central sulcus; CiS – cingulate sulcus; ArS – arcuate sulcus; PS – principal sulcus; a: anterior; p: posterior; d: dorsal; v: ventral.

A mask was created to examine hierarchical clustering within the insula. This insular mask was defined on monkey M12’s anatomical template (both left and right hemisphere) as the area enclosed by the upper and lower bank of the lateral sulcus (Figures 1A, 2A, dashed outline; Supplementary Figure S1). Before performing the hierarchical clustering analysis, we applied bandpass filtering between 0.0025 and 0.05 Hz on the awake resting-state fMRI data (Vincent et al., 2007; Mantini et al., 2011; Sharma et al., 2021). Next, a hierarchical clustering analysis similar to the one described in previous studies (Hutchison and Everling, 2014; Schaeffer et al., 2019a,b; Sharma et al., 2021) was performed. For this, we extracted the time courses of each voxel included in the mask per run. These time courses were then used to calculate the partial correlation between each voxel with every other voxel of that mask, regressing out the mean white matter and ventricles time courses as well as the three-dimensional head motion parameters. After Fisher transforming the correlations to z-scores, we averaged them across runs for each subject after which we averaged across subjects to obtain a final average z-score matrix of all voxels within the predefined mask. Subsequently, we extracted the pairwise standard Euclidean distance between all voxels (using the ‘pdist’ function in MATLAB) in order to convert the average z-score matrix into a distance matrix. This distance gives an indication regarding the voxel to voxel dissimilarity in terms of their temporal correlations. Ultimately, the distance matrix was used as input for the unweighted average-linkage hierarchical clustering analysis (using the ‘linkage’ function in MATLAB) to define the functional cluster solutions of the insular mask. As hierarchical clustering does not require any *a priori* assumptions regarding optimal number of clusters, we conducted the analysis for two- to ten-cluster solutions. Finally, the obtained functional clusters were shown on a flatmap of monkey M12’s brain template using Caret software (version v5.65). In general, finding an optimal number of clusters best reflecting the underlying brain regional organization is not straightforward (Eickhoff et al., 2015) and clustering studies typically use different methods to evaluate the outcome of the clustering algorithms. In order to select the optimal cluster solution in our study, the clusters within each cluster solution were subjected to three different validation methods, including seed-based fingerprinting (Schaeffer et al., 2020; Sharma et al., 2021), the silhouette and elbow method.

### 2.7 Fingerprinting

To determine for which cluster solution the clusters were significantly different from each other regarding their extrinsic intrahemispheric interareal connectivity (or ‘fingerprints’, Mars et al., 2016), we selected 23 predefined spheres of 2 mm radius in each hemisphere as seeds located in areas functionally and/or structurally connected with the insula but external to the defined insular mask (Supplementary Figure S2 shows the location of the 23 left hemisphere seeds). A subsection of these seeds was previously used in a seed-to-brain resting-state study as well as functional clustering study (Sharma et al., 2019, 2021). These regions/seeds include the medial and lateral portion of the primary motor cortex (F1), primary somatosensory cortex (S1) and secondary somatosensory cortex (S2) as well as the dorsal and ventral portion of area F4. Furthermore, we included different subfields of the ventral premotor cortex, including F5c (medial and lateral portion), F5p and F5a (Belmalih et al., 2009; Gerbella et al., 2011; Nelissen and Vanduffel, 2011; Maranesi et al., 2012; Nelissen et al., 2018). In addition, seeds were selected in parietal regions PF, PFG and AIP and in three regions of the frontal operculum: DO, PrCO and GrFO (convexity) (Gerbella et al., 2016; Di Cesare et al., 2019). Based upon previously described ingestive and grasping networks in the orbitofrontal cortex (Borra et al., 2011; Jezzini et al., 2015; Di Cesare et al., 2019; Evrard, 2019) and local maxima of previously published functional gustatory or grasping data from our research group (Sharma et al., 2018, 2019), we selected two seeds in areas 12o and 12r, respectively. Based on their role in processing dynamic social stimuli (Bognár et al., 2023; Cui et al., 2023), we defined two seeds in the superior temporal sulcus (STS), including area FST in the fundus of the superior temporal and neighbouring area MT in the posterior bank of the STS. Previous monkey resting-state fMRI studies suggested functional correlations of the insula with these STS regions (Touroutoglou et al., 2016; Sypré et al., 2023). Finally, a seed was selected in the amygdala due to its structural connections with the insula and its role in encoding emotions and integrating reward and memory with behavior (Amaral and Price, 1984; Stefanacci and Amaral, 2000; Jezzini et al., 2015; Evrard, 2019).

Subsequently, we performed a seed (cluster)-to-seed correlation analysis per run in which we first bandpass-filtered the data and regressed out white matter, ventricle signals and head motion parameters (Vincent et al., 2007; Mantini et al., 2011; Sharma et al., 2019). Next, we correlated the mean time course of each cluster (average of all voxels within a cluster) to the mean time course of each of the external seeds (similar to the functional connectivity analysis described above). The resulting correlation matrix was converted to a z-score matrix by Fischer’s r-to-z transformation after which they were averaged across runs per monkey. This average z-score matrix contains the so-called fingerprint of a cluster per monkey, i.e., the correlation of a cluster with the external seeds (Mars et al., 2016; Schaeffer et al., 2020; Sharma et al., 2021). The average z-scores per cluster (or fingerprints) were subsequently normalized to a range from zero to one and pairwise compared between clusters by calculating the cosine similarity between the normalized fingerprints of the clusters. This similarity measure indicates how similar or different the fingerprint is and can range from −1 to 1 for opposed or identical fingerprint, respectively. Finally, we used a permutation testing approach to test for which cluster solution the cluster fingerprints were still significantly different at the subject level by the use of an in-house code written in MATLAB. First, we randomly shuffled the pairwise cluster labels per monkey, after which we averaged the fingerprints across monkeys (separately for each cluster label), normalized the average fingerprints between 0 and 1 and calculated the cosine similarity. This process was iterated 100,000 times to estimate the distribution of cosine similarities given a null hypothesis that the compared clusters have the same fingerprint. A value of *p* <0.05 (i.e., > 95% of the permutation cosine similarities were larger than the observed value) indicated significantly different fingerprints. A detailed overview of *p*-values for all cluster pairwise comparisons can be found in Supplementary Tables S1–S18.

### 2.8 Additional validation methods

As a second validation method to select the optimal cluster solution, we computed the average silhouette value coefficient (Rousseeuw, 1987). This measure ranges from −1 to 1 and represents the difference between the average distance of a voxel with other voxels of the same clusters compared to the average distance with voxels in other neighbouring clusters. Values approximating one indicate that the voxel is correctly assigned to a cluster while a silhouette value close to zero indicates the voxel could also be assigned to a neighbouring cluster and values close to −1 imply that the voxel was misclassified to a certain sub-cluster. Subsequently, more optimal cluster solutions are associated with higher silhouette values (or at least no significant decrease of silhouette value relative to the K-1 cluster solution). We performed the silhouette analysis using the ‘evalclusters’ function of MATLAB.

Finally, we used the elbow method as a third validation method using an in-house code written in MATLAB. This analysis uses the total within-cluster sum of squares (i.e., a measure of the explained variance within a cluster solution) to assess the optimal number of clusters. The so-called ‘elbow’ determines the cut-off point or optimal cluster solution as for higher cluster solutions the total within-cluster sum of squares decreases in a more linear fashion.

### 2.9 Seed-to-brain functional connectivity analysis

Similar to the analyses described above, we performed band-pass filtering (0.0025–0.05 Hz) as well as regression of white matter, ventricles and head motion parameters prior to determining the mean representative time course by averaging the signal across all voxels that resided within an individual cluster (Vincent et al., 2007; Mantini et al., 2011; Sharma et al., 2019). In order to examine the functional connectivity of individual clusters with the entire brain, Pearson correlations were calculated between the signal of the clusters and the remaining voxels of the brain. These resulting whole-brain connectivity maps were subsequently converted to z-scores using Fisher’s r-to-z-transformation. Finally, fixed-effect analysis was used to generate group-level correlation maps, which were thresholded at z > 2.3 and displayed on a flattened representation of monkey M12’s anatomical template using Caret software (Mantini et al., 2011; Sharma et al., 2019, 2021).

### 2.10 General linear model analysis of the task-based fMRI experiments

A general linear model (GLM) was used to estimate the response amplitude at each voxel (SPM12) following previously described procedures (Friston et al., 1994; Vanduffel et al., 2001). For the awake contrast-enhanced fMRI experiments, this involved convolving a MION hemodynamic response function with a boxcar model to represent the stimulus conditions (Vanduffel et al., 2001). For these experiments, we included nine regressors of no interest in the GLM model (to account for artefacts due to head and eye movements). These nine regressors corresponded to three rotations and three translations along x-, y- and z-axis (head motion) as well as to the horizontal and vertical component of the eye movement and pupil diameter. For each run, this GLM fitting resulted in a map of beta estimates (regression weights) for each condition of interest and for the nine regressors of no interest. GLM analyses were initially performed for each subject separately. Subsequently, the single subject GLM results were pooled together in a fixed-effects group analysis (*n* = 2). Since the galvanic vestibular stimulation data were acquired using BOLD measurements in anesthetized animals, the corresponding BOLD hemodynamic response function was employed and only motion regressors (three rotations and three translations along x-, y- and z-axis) were included in the GLM (Sypré et al., 2023).

### 2.11 Functional response properties of resting-state clusters

In order to characterize the clusters in terms of their functional significance, we used the clusters as regions-of-interest (ROIs) on the different task localizers described in Sypré et al. (2023). For this, we defined each subcluster within the two- to ten-cluster parcellation schemes as an independent ROI. In terms of the visual facial expression localizer, we restricted our analysis to visual responsive voxels (i.e., voxels activated by lip-smacking and scrambled conditions compared to fixation baseline) within each subcluster. For each task, percent MR signal changes (average from all voxels within ROI) were calculated per run within each ROI (subcluster) for each condition of interest against their low-level control condition (respectively distilled water for taste localizer, low concentration sour for distaste/disgust localizer, reach-only for grasping localizer, no stimulation for galvanic stimulation experiment and scrambled faces for facial expression localizer) using MarsBaR (MarsBaR, region-of-interest toolbox for SPM; RRID:SCR_009605). Mean percent signal changes were subsequently determined along with the standard error of the mean. Statistical significance was assessed by one-tailed t-tests where *p*-values were corrected for multiple comparisons (number of subclusters) using false discovery rate (FDR). Ultimately, p-values less than 0.05 after FDR correction were considered significant. A detailed overview of p-values after FDR correction can be found in Supplementary Tables S19, S20 for left and right hemisphere, respectively. *p*-values without any correction applied are represented in Supplementary Tables S21, S22 for left and right hemisphere, respectively.

## 3 Results

### 3.1 Hierarchical clustering of the insular cortex

Since the number of clusters for hierarchical clustering does not need to be set *a priori* (Hutchison and Everling, 2014; Eickhoff et al., 2015) and our aim was exploratory, we conducted the hierarchical clustering analysis for two- to ten-cluster solutions. The resulting parcellation schemes of the hierarchical clustering for two- to ten-cluster solutions in the left and right hemisphere are shown in Figures 1C, 2B, respectively. To select an optimal number of clusters that could best represent the underlying functional specialization of the insula for the current dataset, we examined three commonly used cluster validation methods (fingerprinting, silhouette and elbow).

As a first validation method, we used the fingerprinting technique employed in previous resting-state hierarchical clustering studies (Schaeffer et al., 2020; Sharma et al., 2021). This method consists of a seed-based connectivity analysis of the sub-clusters to identify the cluster solution beyond which sub-clusters can no longer be differentiated in terms of their extrinsic interareal connectivity (Schaeffer et al., 2020; Sharma et al., 2021). The obtained cosine similarity values for the pairwise comparisons of all clusters per cluster solution are represented next to each cluster solution in Figures 1C, 2B for left and right hemisphere, respectively. A permutation testing approach was used to identify cluster solutions in which the clusters were significantly different (*p* < 0.05) from each other in terms of extrinsic interareal connectivity or fingerprints (*cf.* red crosses in the cosine similarity matrices in Figures 1C, 2B for insignificant fingerprints). Corresponding p-values for each pairwise comparison are listed in Supplementary Tables S1–S18. Our permutation testing approach indicated that each sub-cluster was significantly different from all other sub-clusters within the same cluster solution up to the four-cluster solution. Therefore, using this fingerprinting method, the four cluster solution was found optimal for both left (Figure 1) and right (Figure 2) hemispheres. Beginning with the five-cluster solution, the emerging sub-clusters (for example cluster 2 and cluster 3 of the five-cluster solution in left hemisphere; Figure 1C) were no longer significantly different from each other regarding their functional connectivity fingerprints.

The highest average silhouette value was obtained for the two-cluster solution, both for the left hemisphere (Figure 1E) and for the right hemisphere (Figure 2D), suggesting the bipartite cluster solution as most optimal for our current data. However, the average silhouette index also revealed peaks around the four- and five-cluster solutions for both hemispheres, after which this value tended to decrease linearly for more complex (higher number) cluster solutions. This could suggest that the four- and five-cluster solutions also contain valuable information that cannot be deducted from the two-cluster solution. Finally, a third cluster validation criterion we used is the elbow method, which relies on the total within-cluster sum of squares as a measure of the variance within a cluster solution. The location of the bend (elbow) is considered as the optimal number of clusters, beyond which further subdividing the cluster solution does not significantly improve the explained variance. Figures 1F, 2E show all total within-cluster sum of squares for the two- to ten-cluster solutions in the left and right hemisphere, respectively. The elbow method suggested the four- and the five- cluster solutions as most optimal.

Given the outcome of the three different cluster validation methods and the consensus between them, we selected the four-cluster solution as the most optimal parcellation scheme for our current dataset. For the left hemisphere, the four-cluster solution comprised of a large cluster in the anterior portion of the insula, a smaller cluster in the middle dorsal part of the insula, a dorsal-posterior cluster and finally a mid-ventral cluster (Figure 1C). For the right hemisphere, spatial arrangement of the clusters was slightly different, particularly regarding the anterior part of the insula. The four-cluster partitioning scheme for the right hemisphere yielded a posterior cluster, a mid-ventral cluster, a dorsal-anterior and a dorsal-ventral cluster (Figure 2B). Next, we examined the functional response properties and brain-wide functional connectivity to determine the extent to which these four resting-state derived clusters reflect functionally distinct subregions.

### 3.2 Functional properties of insula clusters

**Figure 3:**
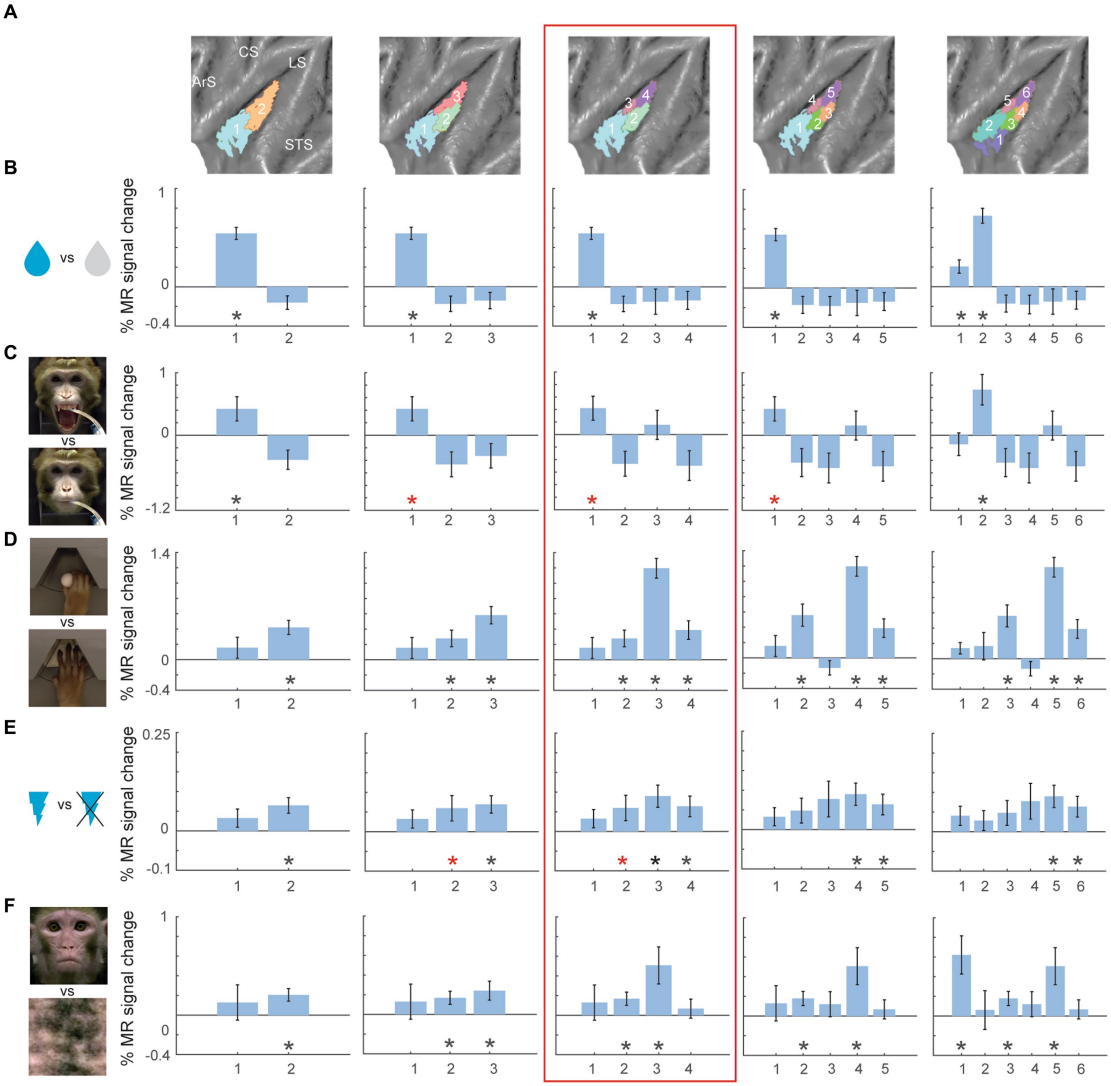
Univariate task-related fMRI responses for two- to six-cluster parcellation schemes in left hemisphere. **(A)** Overview of the two- to six-cluster solutions resulting from the hierarchical clustering analysis. Within each cluster solution, different colors represent individual clusters used as ROIs when calculating the percent signal change. **(B–F)** Percent signal change for taste (sweet and sour flavour) vs. distilled water **(B)**, high concentrated sour liquid vs. low concentrated sour **(C)**, reach-and-grasp execution vs. reach-only execution **(D)**, galvanic vestibular stimulation vs. no stimulation **(E)** and observation of lip-smacking face gestures vs. scrambled dynamic stimuli **(F)** plotted in individual clusters. The numbers on x-axis correspond to the labels of individual clusters in **(A)**. Percent signal changes were calculated for fixed-effects group results (*n* = 2). Error bars indicate standard error of the mean across runs. Black asterisks indicate significant stronger responses for the task condition compared to its corresponding baseline (p < 0.05, one-tailed t-test) after FDR correction. Red asterisks indicate significant responses (p < 0.05, one-tailed t-test) without FDR correction. STS – superior temporal sulcus; LS – lateral sulcus; CS – central sulcus; ArS – arcuate sulcus; a: anterior; p: posterior; d: dorsal; v: ventral.

**Figure 4:**
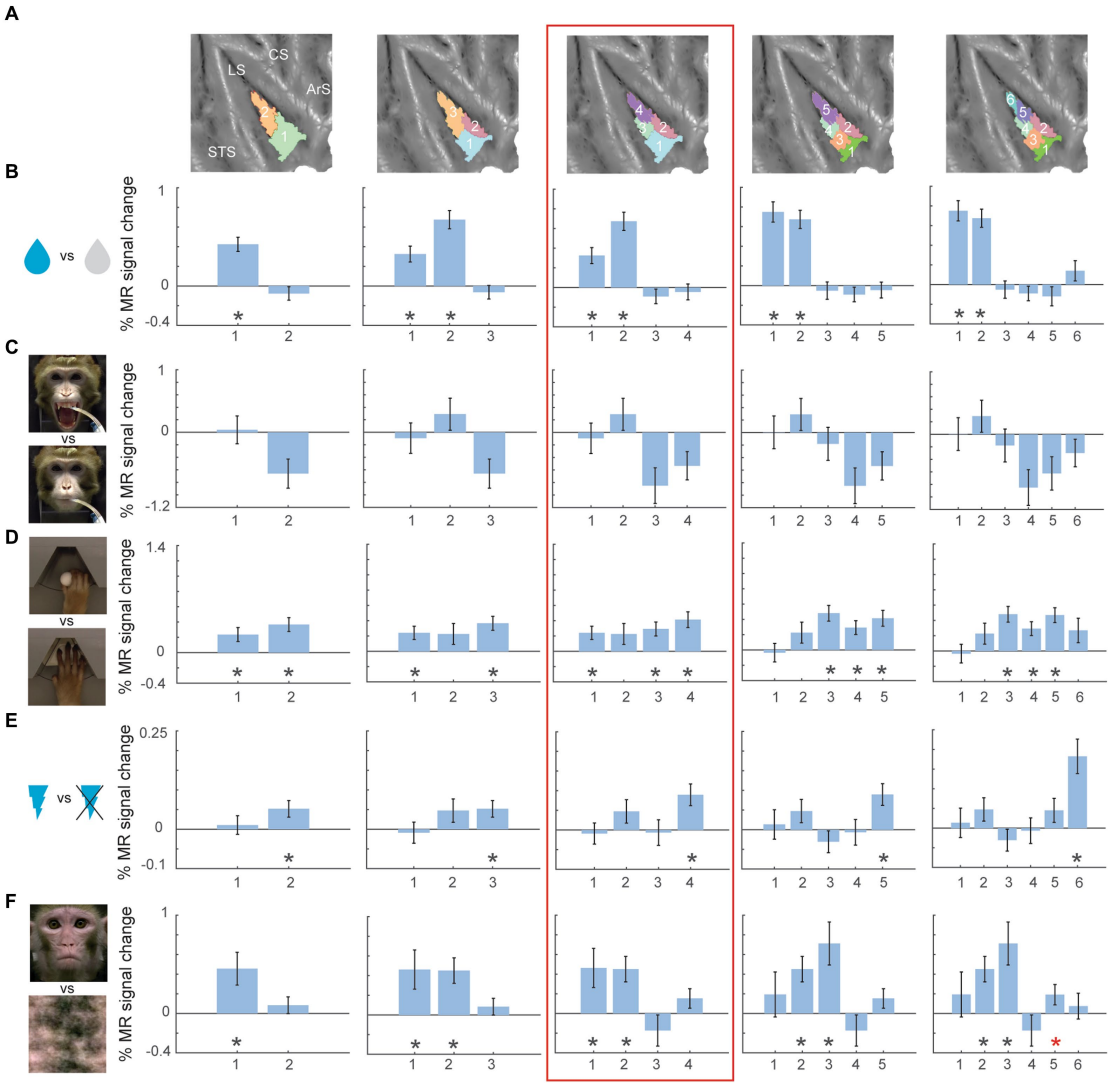
Univariate task-related fMRI responses for two- to six-cluster parcellation schemes in right hemisphere. **(A)** Overview of the two- to six-cluster solutions resulting from the hierarchical clustering analysis. Within each cluster solution, different colors represent individual clusters used as ROIs when calculating the percent signal change. **(B–F)** Percent signal change for taste (sweet and sour flavour) vs. distilled water **(B)**, high concentrated sour liquid vs. low concentrated sour **(C)**, reach-and-grasp execution vs. reach-only execution **(D)**, galvanic vestibular stimulation vs. no stimulation **(E)** and observation of lip-smacking gestures vs. scrambled dynamic stimuli **(F)** plotted in individual clusters. The numbers on x-axis correspond to the labels of individual clusters in **(A)**. Percent signal changes were calculated for fixed-effects group results (*n* = 2). Error bars indicate standard error of the mean across runs. Black asterisks indicate significant stronger responses for the task condition compared to its corresponding baseline (*p* < 0.05, one-tailed t-test) after FDR correction. Red asterisks indicate significant responses (*p* < 0.05, one-tailed t-test) without FDR correction. STS – superior temporal sulcus; LS – lateral sulcus; CS – central sulcus; ArS – arcuate sulcus; a: anterior; p: posterior; d: dorsal; v: ventral.

To investigate the functional response properties of the clusters, we leveraged a range of functional task localizers used previously to characterize the functional organization of the macaque insula (Sypré et al., 2023). These functional localizers involved taste and distaste/disgust processing, hand grasping execution, galvanic vestibular stimulation and observation of lip-smacking facial expressions (Sypré et al., 2023). Figures 3, 4 show the percent MR signal change plots for the two- to six-cluster solutions for the left (Figure 3) and right (Figure 4) hemisphere (response profile for the four cluster solution is highlighted in red box). Functional response profiles for higher cluster number solutions (7 to 10 clusters) for left and right hemisphere are shown in Supplementary Figures S3, S4 respectively. For easier comparison of functional responses in distinct insular subregions across different cluster solutions and hemispheres, in Figures 3, 4 (as well as Supplementary Figures S3, S4), clusters were labelled from low to high numbers along the anterior to posterior axis of the insula. For the left hemisphere, gustatory responses (tastants vs. distilled water or high sour vs. low sour) were mainly observed in the anterior insular clusters (Figures 3B,C). Somato-motor related responses during right hand grasping (compared to reach-only) yielded strongest percent signal changes in the middle dorsal cluster (cluster 3 in the four-cluster solution) (Figure 3D). Galvanic stimulation resulted in significant differential responses (compared to no stimulation) in the dorsal posterior clusters (Figure 3E). Finally, observation of conspecific lip-smacking gestures (compared to phase-scrambled controls) yielded significant MR responses in mid-dorsal and ventral clusters in the four-cluster solution (Figure 3F). Although the three cluster validation criteria (fingerprinting, silhouette and elbow) suggested the four-cluster solution as most optimal, inspecting functional response profiles in cluster parcellations with higher cluster numbers suggests an even more fine-scaled functional differentiation in the insula. For instance while the four-cluster solution suggests predominantly taste responses in the anterior insula (cluster 1 in the four-cluster solution), the six-cluster solution suggests a further differentiation with taste responses mostly driving the upper/middle portion of anterior insula (cluster number 2 in six-cluster solution) and visual responses to social stimuli in the ventral anterior insula (cluster 1 in six-cluster solution).

For the right hemisphere four-cluster solution (Figure 4, red box), taste responses were also strongest in the anterior clusters (Figure 4B). Ipsilateral hand grasping-related responses were present in the posterior dorsal cluster and the two ventral clusters (Figure 4D). Galvanic vestibular stimulation yielded significant responses in the most posterior cluster of the right hemisphere four-cluster parcellation (Figure 4E). Finally, observation of emotional facial expressions (lip-smacking) yielded significant responses in anterior dorsal and ventral clusters (clusters 1 and 2 in the four-cluster solution, Figure 4F).

Like the left hemisphere, inspecting higher cluster dimensions (*N* = 6), suggested an even more fine-grained functional specialization in the right hemisphere. For the six-cluster parcellation, galvanic vestibular stimulation yielded significant responses in particular in the most posterior end of the right insula (cluster 6 in six-cluster solution), while grasping motor responses were found more anterior in dorsal (cluster 5 in six-cluster solution) and ventral (clusters 3 and 4 in six-cluster solution) insula.

### 3.3 Brain-wide functional connectivity of insula clusters

**Figure 5:**
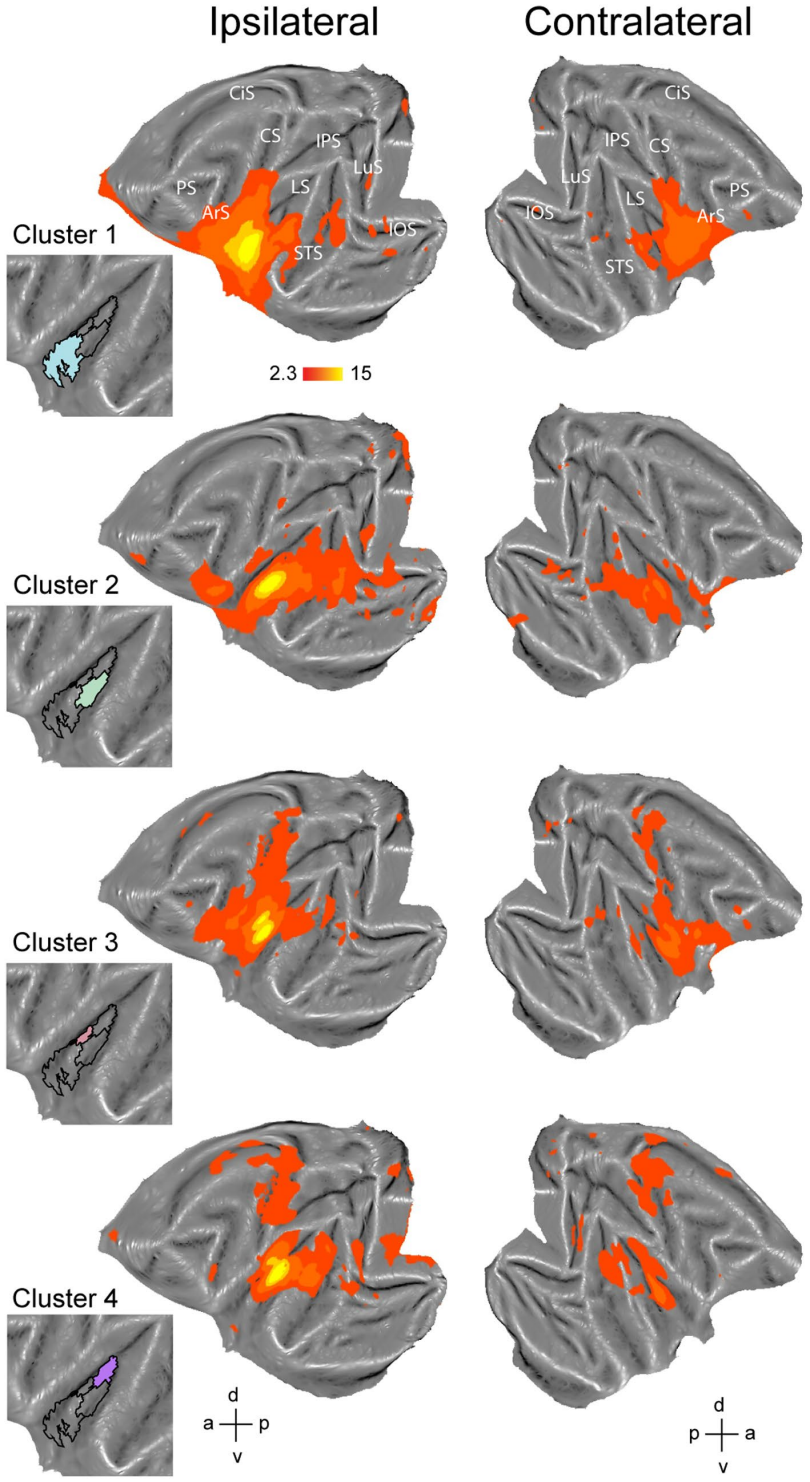
Functional connectivity of the four-cluster solution in left hemisphere. Whole-brain functional connectivity of clusters corresponding to anterior insula (cluster 1), middle part of ventral insula (cluster 2), middle part of dorsal insula (cluster 3) and posterior insula (cluster 4). Insets indicate the cluster that served as seed to calculate the functional connectivity maps. Maps are thresholded at z > 2.3. LuS – lunate sulcus; IOS – inferior occipital sulcus; STS – superior temporal sulcus; LS – lateral sulcus; IPS – intraparietal sulcus; CS – central sulcus; CiS – cingulate sulcus; ArS – arcuate sulcus; PS – principal sulcus; a: anterior; p: posterior; d: dorsal; v: ventral.

**Figure 6:**
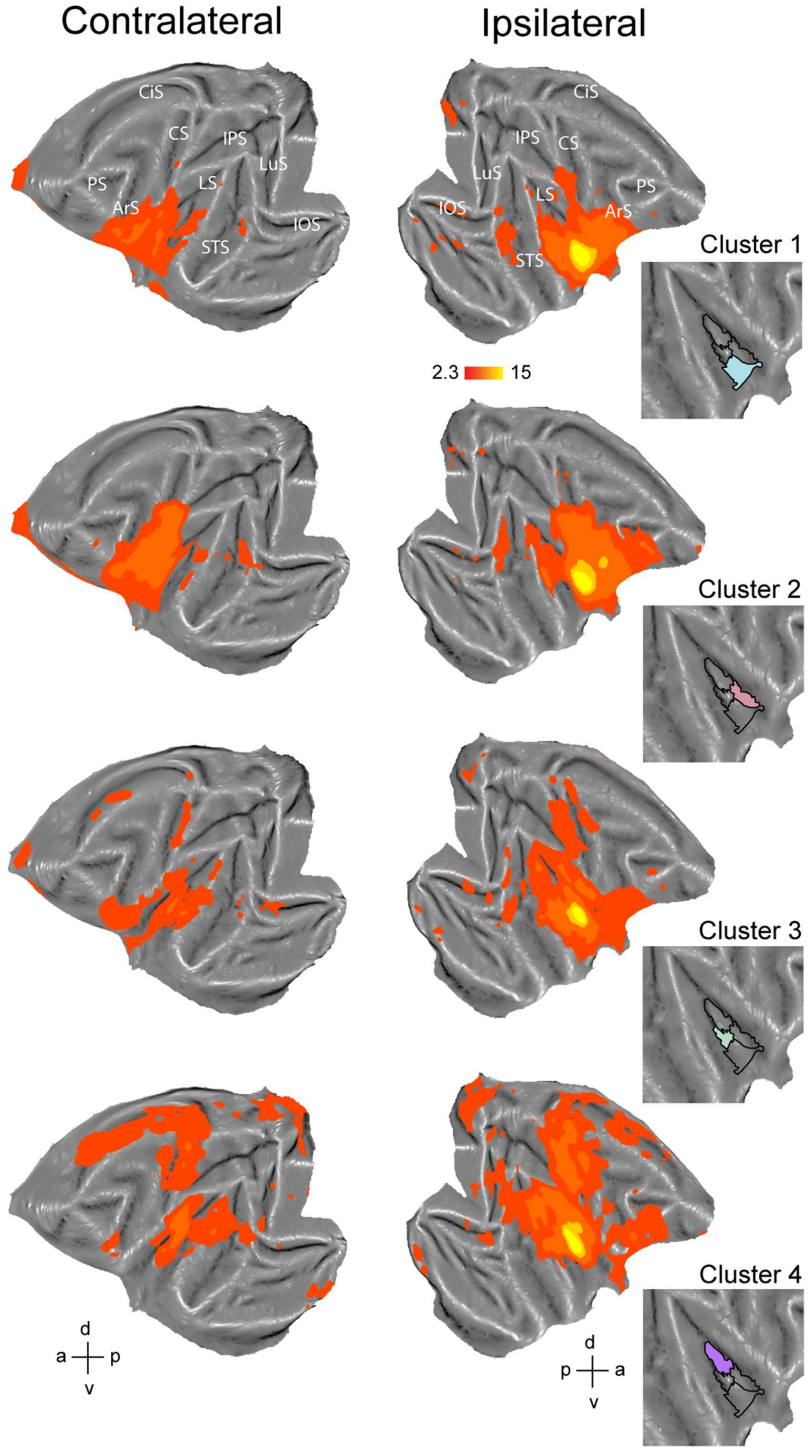
Functional connectivity of the four-cluster solution in right hemisphere. Whole-brain functional connectivity of clusters corresponding to ventral part of anterior insula (cluster 1), middle to anterior part of dorsal insula (cluster 2), middle part of ventral insula (cluster 3) and posterior insula (cluster 4). Insets indicate the cluster that served as seed to calculate the brain correlation maps. Maps are thresholded at z > 2.3. LuS – lunate sulcus; IOS – inferior occipital sulcus; STS – superior temporal sulcus; LS – lateral sulcus; IPS – intraparietal sulcus; CS – central sulcus; CiS – cingulate sulcus; ArS – arcuate sulcus; PS – principal sulcus; a: anterior; p: posterior; d: dorsal; v: ventral.

To further characterize the clusters from the four-cluster solution, we also investigated brain-wide functional connectivity of these four clusters in the left (Figure 5) and right hemisphere (Figure 6) with the rest of the brain. For the left hemisphere, the most anterior cluster (Figure 5, cluster 1, blue) yielded functional connectivity with gustatory- and mouth-related regions including orbitofrontal cortex, frontal opercular areas (GrFO, PrCO, DO), ventral parts of premotor region F4 and F5, ventral portions of F1 and somatosensory areas S1 and S2, inferior parietal area PF and portions of posterior/middle STS and early visual regions. The mid-ventral cluster in the left hemisphere (Figure 5, cluster 2, green) showed functional connectivity with early visual regions, both banks and fundus of posterior and middle STS and lateral sulcus. In addition, this cluster yielded functional connectivity with frontal regions including GrFO and portions of area 12. The smaller mid-dorsal cluster (Figure 5, cluster 3, red) showed functional connectivity with portions of S1, S2, F1, premotor area F5, area 44, area 45, area 46v, parietal areas AIP and PE, as well as posterior areas of the posterior STS. The fourth cluster located in the posterior portion of the left insula (Figure 5, cluster 4, purple) yielded functional correlations with S1, S2, F1, portions of cingulate cortex, dorsal premotor F2 and F3, early visual areas as well as posterior areas of STS including area MST in the upper bank of the STS. In addition, functional correlations were also observed with regions related to processing vestibular and optic flow information (Cottereau et al., 2017), including visual posterior sylvian area (VPS) and putative monkey cingulate sulcus visual area (pmCSv).

The four-cluster solution for the right hemisphere consisted of a posterior, mid-ventral, dorsal anterior and ventral anterior cluster (Figure 6). The dorsal anterior cluster (Figure 6, cluster 2, red) showed functional correlations with orbitofrontal cortex, areas 44, 45 and 46v, premotor F4 and F5, area F1, frontal opercular areas (PrCO, DO and GrFO), somatosensory areas S1 and S2, anterior inferior parietal regions, lower bank of posterior/middle STS and early visual regions. The ventral anterior cluster (Figure 6, cluster 1, blue) showed to a large extent similar functional connectivity as the dorsal anterior cluster. This ventral anterior cluster (Figure 6, cluster 1, blue) showed weaker functional connectivity with ventral F1, frontal operculum and ventral prefrontal areas as compared to the dorsal anterior insular cluster. The mid-ventral cluster in the right hemisphere (Figure 6, cluster 3, green) showed functional correlations with portions of frontal cortex and frontal operculum, early visual cortex, posterior/middle STS and lateral sulcus. This cluster also showed functional connectivity with dorsal regions around the central sulcus including somatosensory and primary motor cortices. Finally, the posterior insular cluster in the right hemisphere (Figure 6, cluster 4, purple) showed functional connectivity that was largely like to the posterior cluster of the left hemisphere (Figure 5, cluster 4, purple). Specifically, the posterior cluster in the right hemisphere displayed functional connectivity with somatosensory cortices S1 and S2, motor F1, frontal cortex, ventral and dorsal premotor cortex and large portions of cingulate cortex, early visual areas and posterior STS (including area MST in the upper bank). Like the left hemisphere, this posterior insula cluster in the right hemisphere also showed functional correlations with VPS near the posterior end of the lateral sulcus and pmCSv in the cingulate cortex.

## 4 Discussion

Over recent years, connectivity based parcellation techniques have become a popular tool to study the functional organization of the brain using non-invasive imaging methods. Several studies have employed connectivity based parcellation methods to examine the organization of the human insula. Using either diffusion weighted imaging, resting-state functional connectivity or meta-analytic task data, these studies have suggested several parcellation schemes for the human insula, including two, three, four or an even higher number of subdivisions (Kurth et al., 2010a,b; Deen et al., 2011; Cauda et al., 2011, 2012; Cloutman et al., 2012; Jakab et al., 2012; Kelly et al., 2012; Chang et al., 2013; Ryali et al., 2013; Nomi et al., 2016; Tian et al., 2019). The diversity of the proposed optimal cluster solutions derived from these methods highlights the difficulty in establishing a single valid parcellation scheme that accurately reflects the underlying functional organization. Furthermore, this is particularly challenging for heterogeneous regions like the insula. It should also be stressed that clustering methods like the intrinsic hierarchical clustering approach used in this study will always attribute each voxel in the region-of-interest or mask to a specific subcluster, hence imposing strict borders between clusters. However, brain regions can also show gradients instead of clear-cut borders in their architecture, connectivity, or functional response properties (Premereur et al., 2015; Margulies et al., 2016). Indeed, as opposed to parcelling the insula into a number of distinct subregions, a different approach has been to describe the insula in terms of its connectivity gradients (Cerliani et al., 2012; Tian and Zalesky, 2018; Wang et al., 2023).

In a previous study, we employed intrinsic hierarchical clustering using resting-state fMRI data to examine the functional organization of macaque ventral premotor cortex F5 and neighbouring regions in the inferior arcuate sulcus (Sharma et al., 2021). We found that this technique indeed could retrieve a detailed level of organization as suggested by invasive functional and anatomical examinations. However, that study also suggested that interpreting resting-state based cluster solutions as reflecting functionally specialized or distinct (sub)regions can be challenging in the absence of additional invasive evidence on the region’s functional and anatomical organization (especially for solutions with higher cluster numbers). Moreover, the organization of most higher-order brain regions and complex multimodal regions like the insula can be described on multiple scales depending on different defining criteria (cyto-, myelo- and receptor architecture, connectivity, functional responses, gradual vs. more abrupt transitions of features, etc). The outcome of clustering-based parcellations, thus, should be best interpreted as reflecting certain differentiation of the region under investigation at different levels of complexity (Kelly et al., 2012; Eickhoff et al., 2015). In particular, the extent to which resting-state derived cluster parcellations, which are based on functional correlations between time series of fMRI signal fluctuations, reflect true or meaningful functional subdivisions remains unclear (Eickhoff et al., 2015). Therefore, interpreting these resting-state derived clusters in terms of biologically relevant subregions subserving some specialized function (different from their neighbours) should preferably be based upon multimodal criteria derived from different complementary techniques (Kelly et al., 2012; Eickhoff et al., 2015; Glasser et al., 2016).

In this study, we examined parcellations of the macaque insula derived from data-driven hierarchical clustering of resting-state fMRI signals. To further assess the functional significance of the intrinsic hierarchical clustering derived parcellations we examined both functional response properties and brain-wide functional connectivity of the clusters. Given that three different cluster validation methods (fingerprinting, silhouette and elbow) converged on four clusters as the optimal solution for our data, we selected the four-cluster parcellation for further seed-to-brain functional connectivity analysis. Compared to the anatomical connectivity based parcellation map of macaque insula proposed in Jezzini et al. (2015) and Figure 1B, these four clusters in the left hemisphere fall within the anterior insular disgust/ingestive field (cluster 1), the somato-motor field in dorsal mid-to-posterior insula (clusters 3 and 4) and the ventral affiliative field according to Jezzini et al. (2015) (cluster 2). Brain-wide functional connectivity of these four clusters in the left hemisphere (Figure 5) indeed are suggestive of a distinct functional role, with the anterior cluster (cluster 1) showing functional connectivity with mouth motor/gustatory networks, the middle dorsal cluster (cluster 3) with somato-motor grasping networks, posterior cluster (cluster 4) with optic flow/vestibular networks and the ventral cluster (cluster 2) with brain regions processing social and affiliative information (Sypré et al., 2023).

For the right hemisphere, the obtained four-cluster parcellation was different from the left hemisphere, in particular with respect to the anterior insula. Besides a posterior cluster and mid-ventral cluster also present in the left hemisphere four-cluster parcellation map, intrinsic clustering suggested a distinct dorsal and ventral cluster in anterior right insula (as opposed to one larger anterior cluster in the left hemisphere). While this might suggest some level of asymmetry (at least in terms of intrinsic function connectivity) between left and right macaque insula, these findings should be interpreted with caution. As has been shown in previous monkey seed-to-brain functional connectivity studies of the insula (Touroutoglou et al., 2016; Sypré et al., 2023), the dorsal and ventral portions of the anterior insula show distinct function connectivity to the rest of the brain. It is therefore unlikely that the single large anterior insula cluster found in the four-cluster solution of the left hemisphere (cluster 1) would reflect a single homogenous functional region. Indeed, at a slightly higher cluster number (*N* = 6), this larger anterior cluster in left insula breaks down in a separate dorsal and ventral cluster (Figure 1C) that both show distinct functional responses properties related to taste/distaste coding (dorsal cluster) or visual responses to social stimuli (ventral cluster) (Figures 3B,C,F). This functional differentiation between dorsal and ventral anterior insula fits with previous anatomical and functional evidence obtained in monkeys (Caruana et al., 2011; Touroutoglou et al., 2016; Evrard, 2019). Although some functional and structural evidence points to possible asymmetries and lateralization of functions in human and non-human primate insula (Craig, 2005; Kurth et al., 2010a,b; Cerliani et al., 2012; Evrard et al., 2012; Jakab et al., 2012; Kucyi et al., 2012; Zhang et al., 2012; Chiarello et al., 2013; Lee et al., 2014; Biduła and Króliczak, 2015; Kann et al., 2016; Levman et al., 2019; Menon et al., 2020; Xia et al., 2020; Sypré et al., 2023), the extent to which the observed asymmetry in some cluster solutions reflects a real anatomical or functional asymmetry in macaques needs further examination.

Examining the fMRI task-based response properties of the four clusters further suggested a functional differentiation amongst these clusters. In line with previous investigations of the macaque insula (Yaxley et al., 1990; Schneider et al., 1993; Verhagen et al., 2004; Remedios et al., 2009; Chen et al., 2010; Caruana et al., 2011; Jezzini et al., 2012; Ishida et al., 2013; Sharma et al., 2018; Evrard, 2019; Kaskan et al., 2019; Sypré et al., 2023), we found taste/distaste responses in anterior insula clusters (Figures 3B,C, 4B), hand/arm somato-motor responses (Figure 3D) in middle insula (strongest in contralateral mid-dorsal cluster), vestibular responses in posterior insula (Figures 3E, 4E), and visual responses to social information depicting conspecific’s affiliative facial expressions in middle insula (dorsal and ventral) and ventral anterior insula (Figures 3F, 4F).

For the current taste localizer data (same data as in Sypré et al., 2023), distilled water was used as a baseline. There is still debate on which is the most adequate baseline stimulus for taste coding studies. While many taste studies indeed used artificial saliva solutions as baseline, research has shown that these stimuli are not always as ‘taste-neutral’ as claimed (Baines et al., 2021). Furthermore, there is no consensus on what would be the most suited composition of these artificial saliva solutions given that saliva composition varies across individuals (Pytko-Polonczyk et al., 2017). Furthermore, to what extent composition of human artificial saliva is similar to that of non-human primates is another point of discussion. Previous awake monkey fMRI studies examining taste responses in the insula have either used artificial saliva (Sharma et al., 2019) or distilled water (Kaskan et al., 2019; Sypré et al., 2023) as baseline to localize taste responses in the brain.

While we selected the four-cluster parcellation as the most optimal cluster solution for our current data, this rather low-dimensional cluster solution does not reflect the full extent of the complex organization of the macaque insula (Jezzini et al., 2015; Evrard, 2019; Sypré et al., 2023). As proposed previously (Nanetti et al., 2009; Kelly et al., 2012; Eickhoff et al., 2015), there might be several possible or meaningful clustering solutions describing the data at different levels of complexity or detail. Examining the functional response characteristics of the higher cluster number parcellations (Figures 3, 4; Supplementary Figures S3, S4) suggests indeed that these optimal cluster solutions only describe the data at a certain level of detail, not necessarily capturing the full complexity of the region being studied. It would therefore, be of interest to examine the extent to which these higher cluster numbers reflect a meaningful level of organization of the insula by examining their functional response profiles using a wider variety of functional tests than the ones used in this study. Overall, our insula clustering study shows that resting-state based intrinsic hierarchical clustering can provide subdivisions that reflect the functional organization of the region at different levels of detail. Nevertheless, cluster parcellations derived from this method are best interpreted in combination with data obtained through other modalities, to provide a more comprehensive and detailed description of the brain region’s functional organization (Kelly et al., 2012; Eickhoff et al., 2015; Glasser et al., 2016).

## Data availability statement

The raw data supporting the conclusions of this article will be made available by the authors, without undue reservation.

## Ethics statement

The animal study was approved by Animal Ethics Committee (KU Leuven) and Local ethics committee (Toulouse University). The study was conducted in accordance with the local legislation and institutional requirements.

## Author contributions

LS: Conceptualization, Data curation, Formal analysis, Investigation, Visualization, Writing – original draft. SS: Data curation, Investigation, Writing – original draft. DM: Resources, Software, Writing – original draft. KN: Conceptualization, Funding acquisition, Investigation, Supervision, Writing – original draft, Writing – review & editing.
