# Supplementary material for: Intrinsic functional clustering of the macaque insular cortex
Source: Front Integr Neurosci. 2024 Jan 5;17:1272529. doi: 10.3389/fnint.2023.1272529 (PMC10797002; doi:10.3389/fnint.2023.1272529)
Supplement: Supplementary file 1 [file Data_Sheet_1.docx]

Supplementary Material

**Intrinsic functional clustering of the macaque insular cortex.**

**Sypré L**^1,2^**, Sharma S**^1,3^**, Mantini D**^2,4^**,** **Nelissen K**^1,2,*^

*** Correspondence:** Corresponding Author: koen.nelissen@kuleuven.be

# Supplementary Figures and Tables

## Supplementary Figures

**
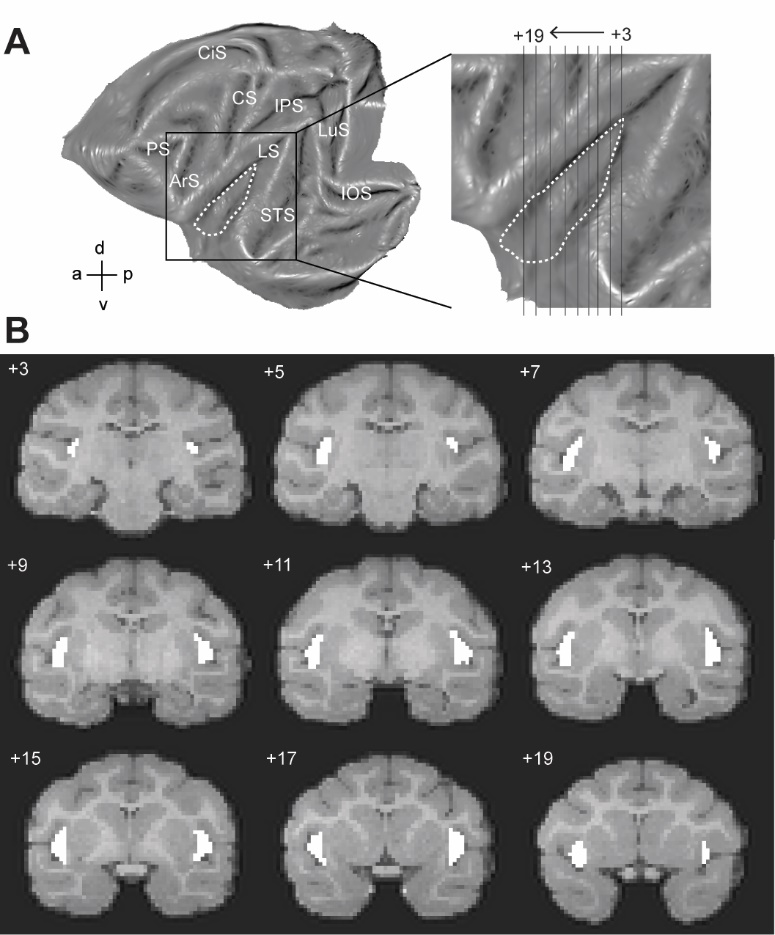
**

**Suppl. Fig. 1. Location of insular ROI used in hierarchical clustering analysis. A.** Representation of insular mask (dashed white line) used in hierarchical clustering analysis on a flattened representation of monkey M12’s anatomical template. Inset indicates the approximate location of the coronal sections shown in B. IOS – inferior occipital sulcus; LuS – lunate sulcus; IPS – intraparietal sulcus; STS – superior temporal sulcus; LS – lateral sulcus; CS – central sulcus; CiS – cingulate sulcus; ArS – arcuate sulcus; PS – principal sulcus; a: anterior; p: posterior; d: dorsal; v: ventral. **B.** Coronal sections indicating the extent of the left and right insula masks used in the hierarchical clustering analysis. Numbers indicate anterior-posterior y-coordinate.


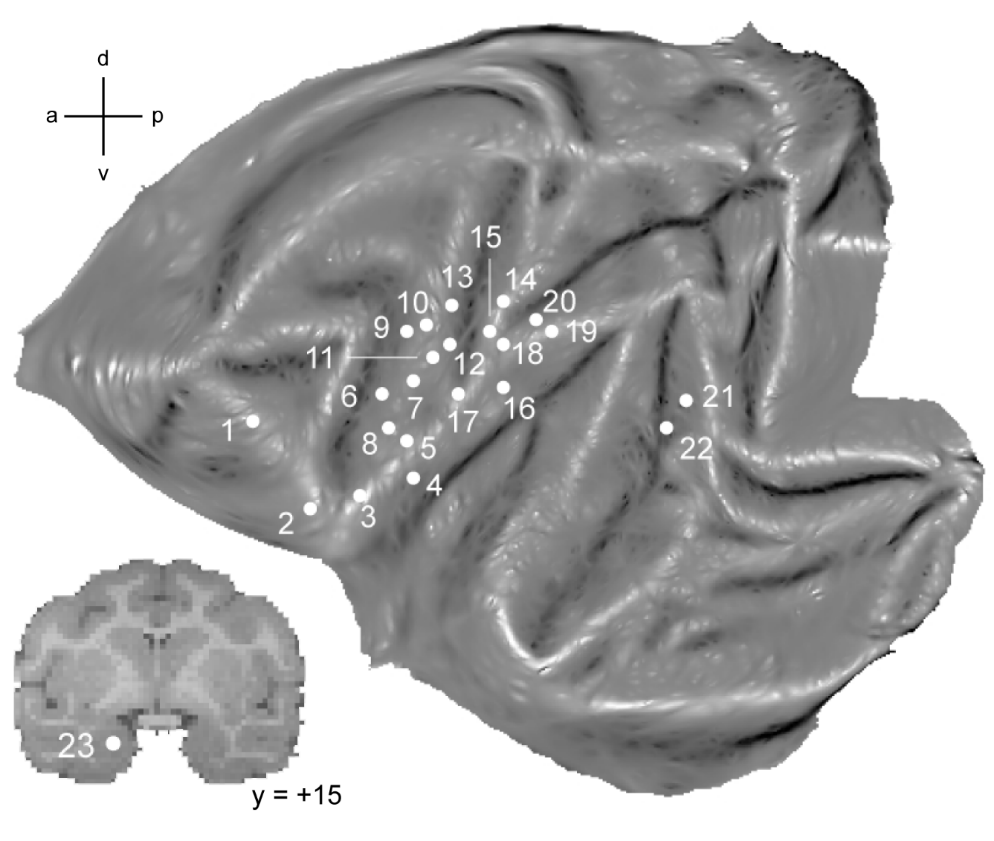


**Suppl. Fig. 2. Location of 23 left hemisphere seeds used for fingerprinting.** 1:12r, 2:12o, 3:GrFO, 4: PrCO, 5: DO, 6: F5a, 7: F5c dorsal, 8: F5c ventral, 9: F5p, 10: F4d, 11: F4v, 12: F1v, 13: F1d, 14: S1med, 15: S1lat, 16: S2med, 17: S2lat, 18: PF, 19: PFG, 20: AIP, 21: MT, 22: FST, 23: amygdala (coronal section, y = +15mm), a: anterior; p: posterior; d: dorsal; v: ventral.

**
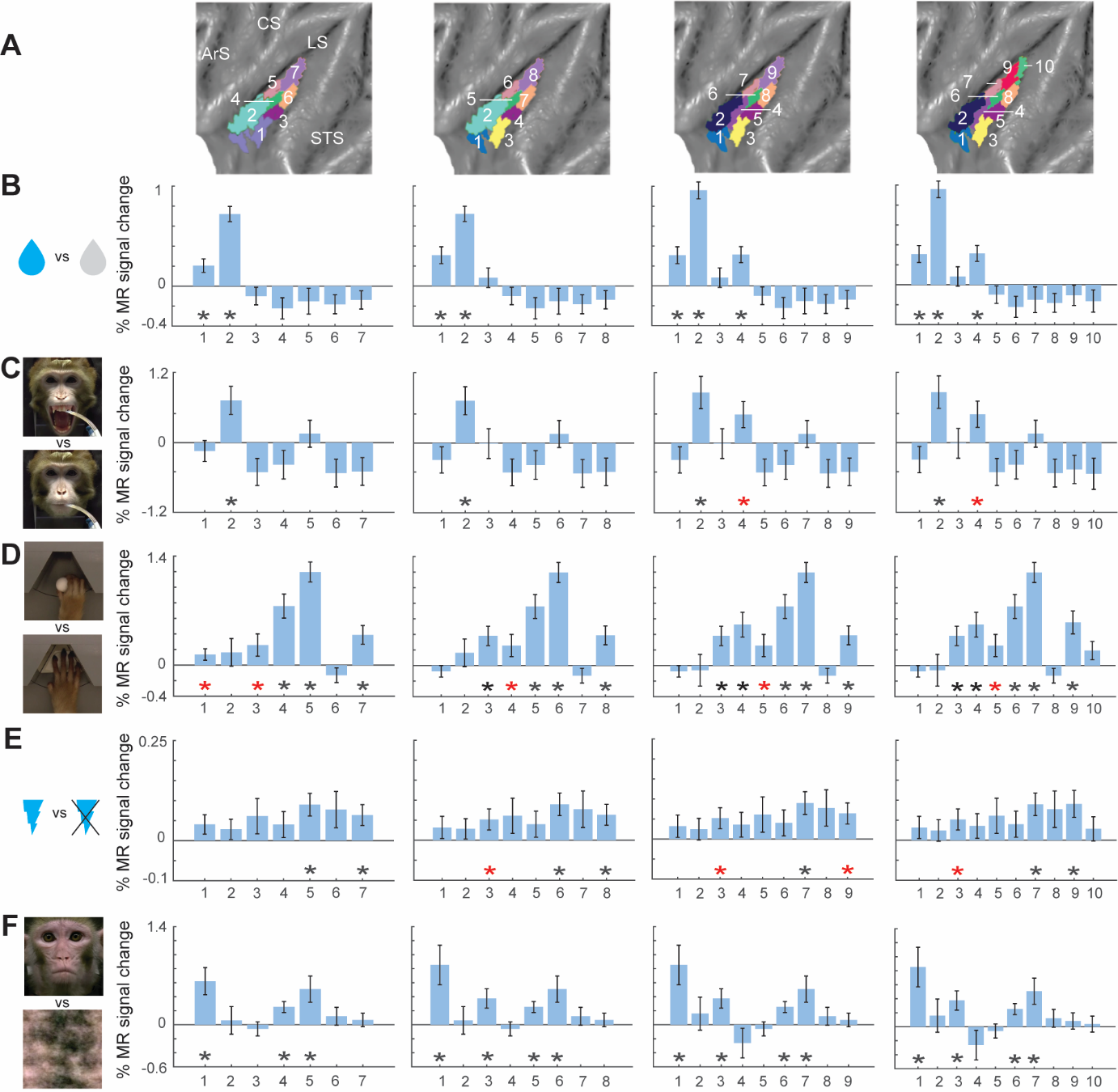
**

**Suppl. Fig. 3. Univariate task-related fMRI responses for seven- to ten-cluster solutions in the left hemisphere. A.** Overview of the seven- to ten-cluster solutions resulting from the hierarchical clustering analysis. Within each cluster solution, different colors represent individual clusters used as ROIs when calculating the percent signal change. **B – F.** Percent signal change for taste (sweet and sour) vs. distilled water (**B**), high concentrated sour liquid vs. low concentrated sour (**C**), reach-and-grasp execution vs. reach-only execution (**D**), galvanic vestibular stimulation vs. no stimulation (**E**) and observation of lip-smacking face gestures vs. scrambled dynamic stimuli (**F**) plotted for individual clusters. The numbers on x-axis correspond to the labels of individual clusters in **A**. Percent signal changes were calculated for fixed-effects group results (n = 2). Error bars indicate standard error of the mean across runs. Black asterisks indicate significant stronger responses for the task condition compared to its corresponding baseline (p < 0.05, one-tailed t-test) after FDR correction. Red asterisks indicate significant responses (p < 0.05, one-tailed t-test) without FDR correction. STS – superior temporal sulcus; LS – lateral sulcus; CS – central sulcus; ArS – arcuate sulcus; a: anterior; p: posterior; d: dorsal; v: ventral.


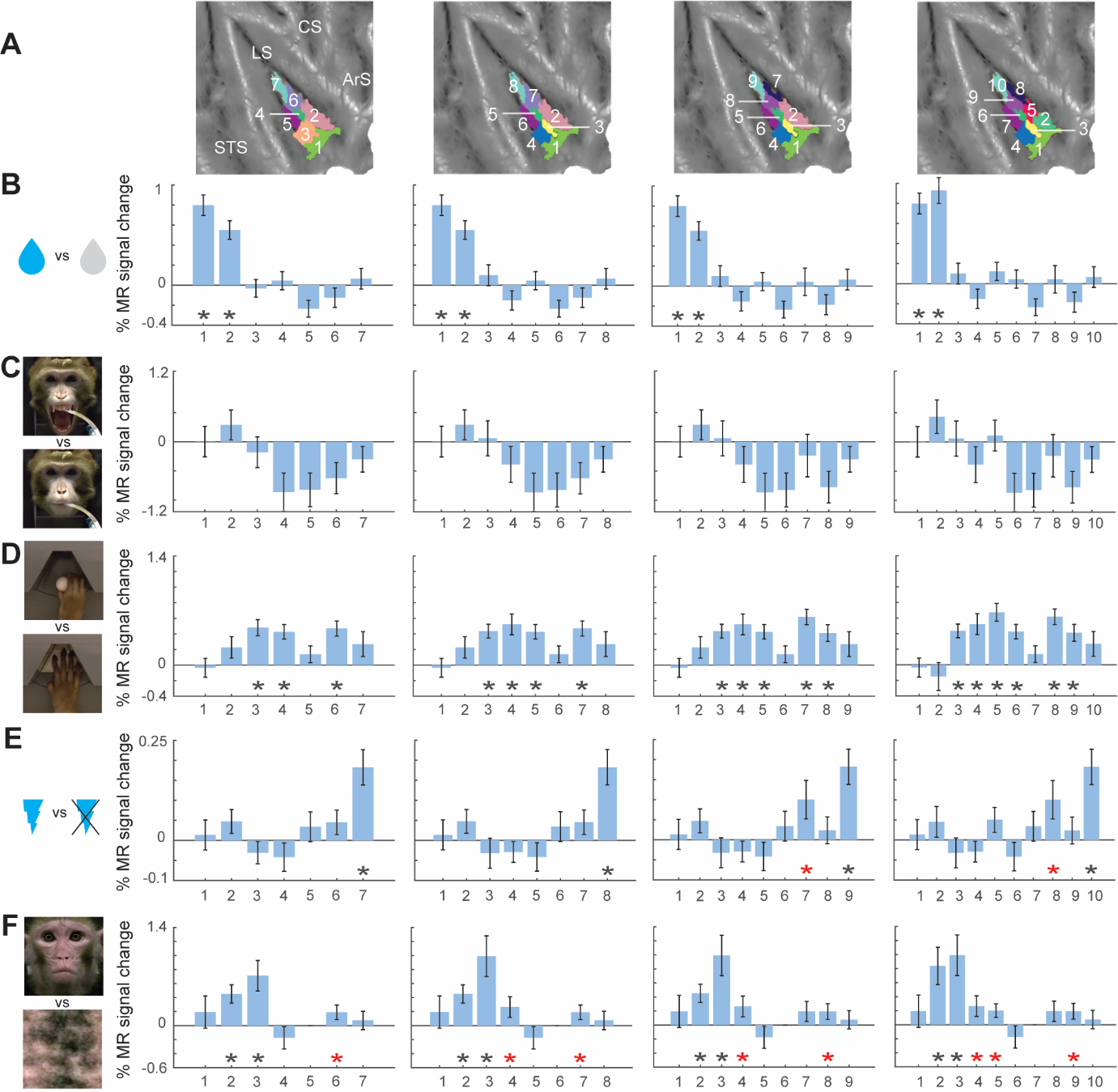


**Suppl. Fig. 4. Univariate task-related fMRI responses for seven- to ten-cluster solutions in right hemisphere. A.** Overview of the seven- to ten-cluster solutions resulting from the hierarchical clustering analysis. Within each cluster solution, different colors represent individual clusters used as ROIs when calculating the percent signal change. **B – F.** Percent signal change for taste (sweet and sour) vs. distilled water (**B**), high concentrated sour liquid vs. low concentrated sour (**C**), reach-and-grasp execution vs. reach-only execution (**D**), galvanic vestibular stimulation vs. no stimulation (**E**) and observation of lip-smacking face gestures vs. scrambled dynamic stimuli (**F**) plotted in individual clusters. The numbers on x-axis correspond to the labels of individual clusters in **A**. Percent signal changes were calculated for fixed-effects group results (n = 2). Error bars indicate standard error of the mean across runs. Black asterisks indicate significant stronger responses for the task condition compared to its corresponding baseline (p < 0.05, one-tailed t-test) after FDR correction. Red asterisks indicate significant responses (p < 0.05, one-tailed t-test) without FDR correction. STS – superior temporal sulcus; LS – lateral sulcus; CS – central sulcus; ArS – arcuate sulcus; a: anterior; p: posterior; d: dorsal; v: ventral.

## Supplementary Tables

| C1 |  |
| --- | --- |
| **0.0076** | C2 |

**Table S1.** P-values for pairwise cluster comparison of the 2-cluster solution in the left hemisphere. P-values were obtained using a permutation testing approach and used to assess the similarity between clusters in terms of their extrinsic interareal connectivity with 23 predefined seeds. The significance level was set at p < 0.05 and p-values in red indicate insignificantly different fingerprints of the compared clusters.

| C1 |  |  |
| --- | --- | --- |
| **0.0080** | C2 |  |
| **0.0076** | **0.0080** | C3 |

**Table S2.** P-values for pairwise cluster comparison of the 3-cluster solution in the left hemisphere. Same conventions as in Table S1.

| C1 |  |  |  |
| --- | --- | --- | --- |
| **0.0076** | C2 |  |  |
| **0.0078** | **0.0078** | C3 |  |
| **0.0079** | **0.0077** | **0.0316** | C4 |

**Table S3.** P-values for pairwise cluster comparison of the 4-cluster solution in the left hemisphere. Same conventions as in Table S1.

| C1 |  |  |  |  |
| --- | --- | --- | --- | --- |
| **0.0080** | C2 |  |  |  |
| **0.0084** | 0.3216 | C3 |  |  |
| **0.0080** | **0.0079** | **0.0076** | C4 |  |
| **0.0075** | **0.0077** | **0.0070** | **0.0314** | C5 |

**Table S4.** P-values for pairwise cluster comparison of the 5-cluster solution in the left hemisphere. Same conventions as in Table S1.

| C1 |  |  |  |  |  |
| --- | --- | --- | --- | --- | --- |
| **0.0076** | C2 |  |  |  |  |
| **0.0305** | 0.3187 | C3 |  |  |  |
| **0.0081** | **0.0080** | **0.0079** | C4 |  |  |
| **0.0082** | **0.0081** | **0.0079** | **0.0079** | C5 |  |
| **0.0077** | **0.0078** | **0.0079** | **0.0074** | **0.0318** | C6 |

**Table S5.** P-values for pairwise cluster comparison of the 6-cluster solution in the left hemisphere. Same conventions as in Table S1.

| C1 |  |  |  |  |  |  |
| --- | --- | --- | --- | --- | --- | --- |
| **0.0084** | C2 |  |  |  |  |  |
| **0.0307** | 0.4848 | C3 |  |  |  |  |
| **0.0079** | **0.0081** | **0.0080** | C4 |  |  |  |
| **0.0229** | **0.0076** | 0.1644 | **0.0079** | C5 |  |  |
| **0.0078** | **0.0078** | **0.0072** | **0.0079** | **0.0231** | C6 |  |
| **0.0084** | **0.0082** | **0.0076** | **0.0080** | **0.0226** | **0.0307** | C7 |

**Table S6.** P-values for pairwise cluster comparison of the 7-cluster solution in the left hemisphere. Same conventions as in Table S1.

| C1 |  |  |  |  |  |  |  |
| --- | --- | --- | --- | --- | --- | --- | --- |
| 0.1733 | C2 |  |  |  |  |  |  |
| 0.0559 | 0.4862 | C3 |  |  |  |  |  |
| **0.0072** | **0.0080** | **0.0072** | C4 |  |  |  |  |
| **0.0316** | **0.0076** | 0.1634 | **0.0081** | C5 |  |  |  |
| **0.0078** | **0.0077** | **0.0152** | **0.0157** | **0.0078** | C6 |  |  |
| **0.0081** | **0.0082** | **0.0082** | **0.0079** | **0.0237** | **0.0158** | C7 |  |
| **0.0080** | **0.0076** | **0.0079** | **0.0078** | **0.0234** | **0.0158** | **0.0303** | C8 |

**Table S7.** P-values for pairwise cluster comparison of the 8-cluster solution in the left hemisphere. Same conventions as in Table S1.

| C1 |  |  |  |  |  |  |  |  |
| --- | --- | --- | --- | --- | --- | --- | --- | --- |
| 0.0556 | C2 |  |  |  |  |  |  |  |
| **0.0073** | **0.0078** | C3 |  |  |  |  |  |  |
| **0.321** | 0.1654 | **0.0077** | C4 |  |  |  |  |  |
| **0.0078** | **0.0080** | **0.0076** | **0.0079** | C5 |  |  |  |  |
| **0.0077** | **0.0153** | 0.1937 | **0.0074** | **0.0077** | C6 |  |  |  |
| **0.0079** | **0.0081** | **0.0080** | **0.0236** | **0.0080** | **0.0160** | C7 |  |  |
| **0.0079** | **0.0079** | **0.0077** | **0.0236** | **0.0078** | **0.0154** | **0.0311** | C8 |  |
| 0.1752 | 0.4862 | **0.0082** | **0.0078** | **0.0075** | **0.0079** | **0.0076** | **0.0082** | C9 |

**Table S8.** P-values for pairwise cluster comparison of the 9-cluster solution in the left hemisphere. Same conventions as in Table S1.

| C1 |  |  |  |  |  |  |  |  |  |
| --- | --- | --- | --- | --- | --- | --- | --- | --- | --- |
| 0.0548 | C2 |  |  |  |  |  |  |  |  |
| **0.0078** | **0.0081** | C3 |  |  |  |  |  |  |  |
| **0.0305** | 0.1618 | **0.0075** | C4 |  |  |  |  |  |  |
| **0.0077** | **0.0076** | **0.0079** | **0.0081** | C5 |  |  |  |  |  |
| **0.0079** | **0.0157** | 0.1934 | **0.0075** | **0.0078** | C6 |  |  |  |  |
| **0.0155** | **0.0465** | **0.0079** | 0.1869 | **0.0076** | **0.0295** | C7 |  |  |  |
| **0.0075** | **0.0078** | **0.0076** | **0.0150** | **0.0081** | **0.0073** | 0.0711 | C8 |  |  |
| 0.1723 | 0.4846 | **0.0081** | **0.0077** | **0.0071** | **0.0082** | **0.0234** | **0.0080** | C9 |  |
| **0.0082** | **0.0079** | **0.0081** | **0.0226** | **0.0080** | **0.0163** | **0.0312** | **0.0240** | **0.0075** | C10 |

**Table S9.** P-values for pairwise cluster comparison of the 10-cluster solution in the left hemisphere. Same conventions as in Table S1.

| C1 |  |
| --- | --- |
| **0.0152** | C2 |

**Table S10.** P-values for pairwise cluster comparison of the 2-cluster solution in the right hemisphere. Same conventions as in Table S1.

| C1 |  |  |
| --- | --- | --- |
| **0.0080** | C2 |  |
| **0.0076** | **0.0161** | C3 |

**Table S11.** P-values for pairwise cluster comparison of the 3-cluster solution in the right hemisphere. Same conventions as in Table S1.

| C1 |  |  |  |
| --- | --- | --- | --- |
| **0.0311** | C2 |  |  |
| **0.0078** | **0.0308** | C3 |  |
| **0.0079** | **0.0157** | **0.0148** | C4 |

**Table S12.** P-values for pairwise cluster comparison of the 4-cluster solution in the right hemisphere. Same conventions as in Table S1.

| C1 |  |  |  |  |
| --- | --- | --- | --- | --- |
| **0.0080** | C2 |  |  |  |
| **0.0233** | 0.0689 | C3 |  |  |
| **0.0080** | **0.0079** | **0.0306** | C4 |  |
| **0.0075** | **0.0161** | **0.0154** | **0.0157** | C5 |

**Table S13.** P-values for pairwise cluster comparison of the 5-cluster solution in the right hemisphere. Same conventions as in Table S1.

| C1 |  |  |  |  |  |
| --- | --- | --- | --- | --- | --- |
| **0.0075** | C2 |  |  |  |  |
| **0.0236** | 0.0694 | C3 |  |  |  |
| **0.0073** | **0.0082** | 0.1329 | C4 |  |  |
| **0.0075** | **0.0153** | **0.0079** | 0.4539 | C5 |  |
| **0.0080** | **0.0155** | **0.0155** | **0.0074** | **0.0165** | C6 |

**Table S14.** P-values for pairwise cluster comparison of the 6-cluster solution in the right hemisphere. Same conventions as in Table S1.

| C1 |  |  |  |  |  |  |
| --- | --- | --- | --- | --- | --- | --- |
| **0.0084** | C2 |  |  |  |  |  |
| **0.0161** | **0.0310** | C3 |  |  |  |  |
| **0.0313** | 0.0544 | **0.0080** | C4 |  |  |  |
| **0.0073** | **0.0076** | 0.1649 | **0.0396** | C5 |  |  |
| **0.0078** | **0.0156** | **0.0312** | **0.0160** | 0.4529 | C6 |  |
| **0.0084** | **0.0161** | **0.0152** | **0.0233** | **0.0080** | **0.0153** | C7 |

**Table S15.** P-values for pairwise cluster comparison of the 7-cluster solution in the right hemisphere. Same conventions as in Table S1.

| C1 |  |  |  |  |  |  |  |
| --- | --- | --- | --- | --- | --- | --- | --- |
| **0.0074** | C2 |  |  |  |  |  |  |
| **0.0159** | 0.1185 | C3 |  |  |  |  |  |
| **0.0381** | **0.0317** | **0.0311** | C4 |  |  |  |  |
| **0.0307** | 0.3049 | **0.0076** | 0.0555 | C5 |  |  |  |
| **0.0078** | 0.1036 | 0.1647 | **0.0082** | **0.0399** | C6 |  |  |
| **0.0081** | **0.0243** | **0.0312** | **0.0163** | **0.0152** | 0.4537 | C7 |  |
| **0.0080** | **0.0154** | **0.0157** | **0.0158** | **0.0227** | **0.0078** | **0.0150** | C8 |

**Table S16.** P-values for pairwise cluster comparison of the 8-cluster solution in the right hemisphere. Same conventions as in Table S1.

| C1 |  |  |  |  |  |  |  |  |
| --- | --- | --- | --- | --- | --- | --- | --- | --- |
| **0.0078** | C2 |  |  |  |  |  |  |  |
| **0.0152** | 0.1159 | C3 |  |  |  |  |  |  |
| **0.0389** | **0.0307** | **0.0305** | C4 |  |  |  |  |  |
| **0.0308** | 0.3064 | **0.0076** | 0.0554 | C5 |  |  |  |  |
| **0.0077** | 0.1183 | 0.2192 | **0.0156** | **0.0383** | C6 |  |  |  |
| **0.0079** | **0.0241** | **0.0310** | **0.0155** | **0.0151** | 0.3500 | C7 |  |  |
| **0.0079** | **0.0155** | **0.0151** | **0.0162** | **0.0230** | **0.0073** | **0.0156** | C8 |  |
| **0.0079** | 0.1019 | 0.1493 | **0.0078** | 0.0545 | 0.1656 | 0.4364 | **0.0082** | C9 |

**Table S17.** P-values for pairwise cluster comparison of the 9-cluster solution in the right hemisphere. Same conventions as in Table S1.

| C1 |  |  |  |  |  |  |  |  |  |
| --- | --- | --- | --- | --- | --- | --- | --- | --- | --- |
| **0.0079** | C2 |  |  |  |  |  |  |  |  |
| **0.0159** | 0.1151 | C3 |  |  |  |  |  |  |  |
| **0.0390** | **0.0311** | **0.0300** | C4 |  |  |  |  |  |  |
| **0.0309** | 0.3039 | **0.0079** | 0.0555 | C5 |  |  |  |  |  |
| **0.0079** | 0.1173 | 0.2189 | **0.0154** | **0.0390** | C6 |  |  |  |  |
| **0.0077** | **0.0231** | **0.0311** | **0.0159** | **0.0152** | 0.3515 | C7 |  |  |  |
| **0.0075** | **0.0078** | **0.0076** | **0.0078** | **0.0311** | **0.0073** | **0.0072** | C8 |  |  |
| **0.0082** | **0.0233** | **0.0157** | **0.0157** | **0.0152** | **0.0082** | **0.0152** | **0.0080** | C9 |  |
| **0.0082** | 0.1010 | 0.1490 | **0.0072** | 0.0546 | 0.1652 | 0.4346 | **0.0081** | **0.0075** | C10 |

**Table S18.** P-values for pairwise cluster comparison of the 10-cluster solution in the right hemisphere. Same conventions as in Table S1.

|  | Task 1 | Task 2 | Task 3 | Task 4 | Task 5 |
| --- | --- | --- | --- | --- | --- |
|  | (Taste vs Distilled water) | (High vs Low conc. sour) | (Grasp vs Reach) | (Vestibular stim. vs No stimulation) | (Lipsmack vs Scrambled stimuli) |
| **Two cluster solution** |  |  |  |  |  |
| Cluster 1 | 3.47 X 10^-12^ | 0.0380 | 0.1420 | 0.0861 | 0.2365 |
| Cluster 2 | 0.9889 | 0.9913 | 7.98 X 10^-4^ | 0.0033 | 0.0019 |
| **Three cluster solution** |  |  |  |  |  |
| Cluster 1 | 5.21 X 10^-12^ | 0.0570 | 0.1420 | 0.0861 | 0.2365 |
| Cluster 2 | 0.9845 | 0.9861 | 0.0203 | 0.0572 | 0.0109 |
| Cluster 3 | 0.9845 | 0.9861 | 4.89 X 10^-4^ | 0.0067 | 0.0109 |
| **Four cluster solution** |  |  |  |  |  |
| Cluster 1 | 6.94 X 10^-12^ | 0.0761 | 0.1420 | 0.0861 | 0.2430 |
| Cluster 2 | 0.9845 | 0.9861 | 0.0180 | 0.0508 | 0.0145 |
| Cluster 3 | 0.9845 | 0.5119 | 3.11 X 10^-6^ | 0.0083 | 0.0145 |
| Cluster 4 | 0.9845 | 0.9861 | 0.0086 | 0.0232 | 0.2430 |
| **Five cluster solution** |  |  |  |  |  |
| Cluster 1 | 8.68 X 10^-12^ | 0.0951 | 0.1774 | 0.0861 | 0.2430 |
| Cluster 2 | 0.9735 | 0.9808 | 0.0033 | 0.0861 | 0.0181 |
| Cluster 3 | 0.9735 | 0.9808 | 0.9046 | 0.0846 | 0.2430 |
| Cluster 4 | 0.9735 | 0.6399 | 3.89 X 10^-6^ | 0.0104 | 0.0181 |
| Cluster 5 | 0.9735 | 0.9808 | 0.0072 | 0.0290 | 0.2430 |
| **Six cluster solution** |  |  |  |  |  |
| Cluster 1 | 0.0055 | 0.9808 | 0.0687 | 0.0817 | 0.0064 |
| Cluster 2 | 9.18 X 10^-13^ | 0.0180 | 0.2284 | 0.1380 | 0.3782 |
| Cluster 3 | 0.9735 | 0.9808 | 0.0040 | 0.0847 | 0.0144 |
| Cluster 4 | 0.9735 | 0.9808 | 0.9046 | 0.0817 | 0.2611 |
| Cluster 5 | 0.9735 | 0.7679 | 4.67 X 10^-6^ | 0.0124 | 0.0135 |
| Cluster 6 | 0.9735 | 0.9808 | 0.0086 | 0.0348 | 0.2916 |
| **Seven cluster solution** |  |  |  |  |  |
| Cluster 1 | 0.0064 | 0.9808 | 0.0698 | 0.0953 | 0.0037 |
| Cluster 2 | 1.07 X 10^-12^ | 0.0211 | 0.2221 | 0.1380 | 0.4413 |
| Cluster 3 | 0.9795 | 0.9808 | 0.0698 | 0.1244 | 0.7243 |
| Cluster 4 | 0.9795 | 0.9808 | 8.18 X 10^-4^ | 0.1343 | 0.0037 |
| Cluster 5 | 0.9795 | 0.8959 | 5.45 X 10^-6^ | 0.0145 | 0.0105 |
| Cluster 6 | 0.9795 | 0.9808 | 0.9046 | 0.0953 | 0.3046 |
| Cluster 7 | 0.9795 | 0.9808 | 0.0100 | 0.0406 | 0.3402 |
| **Eight cluster solution** |  |  |  |  |  |
| Cluster 1 | 0.0012 | 0.9808 | 0.9046 | 0.1380 | 0.0074 |
| Cluster 2 | 1.22 X 10^-12^ | 0.0241 | 0.2538 | 0.1380 | 0.4323 |
| Cluster 3 | 0.5341 | 0.9808 | 0.0111 | 0.0799 | 0.0090 |
| Cluster 4 | 0.9795 | 0.9808 | 0.0798 | 0.1380 | 0.7243 |
| Cluster 5 | 0.9795 | 0.9808 | 9.34 X 10^-4^ | 0.1380 | 0.0074 |
| Cluster 6 | 0.9795 | 0.9808 | 6.23 X 10^-6^ | 0.0166 | 0.0090 |
| Cluster 7 | 0.9795 | 0.9808 | 0.9046 | 0.1015 | 0.2785 |
| Cluster 8 | 0.9795 | 0.9808 | 0.0111 | 0.0464 | 0.3240 |
| **Nine cluster solution** |  |  |  |  |  |
| Cluster 1 | 9.07 X 10^-4^ | 0.9808 | 0.9046 | 0.1477 | 0.0084 |
| Cluster 2 | 2.00 X 10^-15^ | 0.0207 | 0.7895 | 0.1875 | 0.3254 |
| Cluster 3 | 0.4506 | 0.9808 | 0.0100 | 0.0899 | 0.0101 |
| Cluster 4 | 5.08 X 10^-4^ | 0.0902 | 0.0097 | 0.1477 | 0.8897 |
| Cluster 5 | 0.9795 | 0.9808 | 0.0748 | 0.1477 | 0.8148 |
| Cluster 6 | 0.9795 | 0.9808 | 0.0011 | 0.1477 | 0.0084 |
| Cluster 7 | 0.9795 | 0.7679 | 7.01 X 10^-6^ | 0.0187 | 0.0101 |
| Cluster 8 | 0.9795 | 0.9808 | 0.9046 | 0.1142 | 0.3133 |
| Cluster 9 | 0.9795 | 0.9808 | 0.0097 | 0.0522 | 0.3254 |
| **Ten cluster solution** |  |  |  |  |  |
| Cluster 1 | 0.0010 | 0.9808 | 0.9046 | 0.1642 | 0.0093 |
| Cluster 2 | 2.22 X 10^-15^ | 0.0230 | 0.7676 | 0.1875 | 0.3616 |
| Cluster 3 | 0.5007 | 0.9808 | 0.0111 | 0.0999 | 0.0112 |
| Cluster 4 | 5.65 X 10^-4^ | 0.1003 | 0.0090 | 0.1642 | 0.8897 |
| Cluster 5 | 0.9795 | 0.9808 | 0.0831 | 0.1642 | 0.8047 |
| Cluster 6 | 0.9795 | 0.9808 | 0.0012 | 0.1642 | 0.0093 |
| Cluster 7 | 0.9795 | 0.8532 | 7.78 X 10^-6^ | 0.0207 | 0.0112 |
| Cluster 8 | 0.9795 | 0.9808 | 0.9046 | 0.1269 | 0.3481 |
| Cluster 9 | 0.9795 | 0.9808 | 0.0054 | 0.0324 | 0.3616 |
| Cluster 10 | 0.9795 | 0.9808 | 0.0921 | 0.1875 | 0.4620 |

**Table S19.** Results of one-tail one-sample t-tests corrected for multiple comparisons (FDR) for the univariate task-related fMRI responses in the two to ten cluster solution for left hemisphere.

|  | Task 1 | Task 2 | Task 3 | Task 4 | Task 5 |
| --- | --- | --- | --- | --- | --- |
|  | (Taste vs Distilled water) | (High vs Low conc. sour) | (Grasp vs Reach) | (Vestibular stim. vs No stimulation) | (Lipsmack vs Scrambled stimuli) |
| **Two cluster solution** |  |  |  |  |  |
| Cluster 1 | 2.13 X 10^-7^ | 0.8602 | 0.0130 | 0.3305 | 0.0080 |
| Cluster 2 | 0.8640 | 0.9956 | 0.0020 | 0.0217 | 0.1642 |
| **Three cluster solution** |  |  |  |  |  |
| Cluster 1 | 3.34 X 10^-5^ | 0.9730 | 0.0135 | 0.6293 | 0.0176 |
| Cluster 2 | 2.64 X 10^-7^ | 0.4019 | 0.0628 | 0.0930 | 0.0014 |
| Cluster 3 | 0.8640 | 0.9956 | 0.0029 | 0.0326 | 0.1642 |
| **Four cluster solution** |  |  |  |  |  |
| Cluster 1 | 4.45 X 10^-5^ | 0.9968 | 0.0120 | 0.6293 | 0.0235 |
| Cluster 2 | 3.52 X 10^-7^ | 0.5358 | 0.0628 | 0.1239 | 0.0019 |
| Cluster 3 | 0.8694 | 0.9968 | 0.0083 | 0.6293 | 0.8603 |
| Cluster 4 | 0.8694 | 0.9968 | 0.0041 | 0.0071 | 0.0856 |
| **Five cluster solution** |  |  |  |  |  |
| Cluster 1 | 4.45 X 10^-10^ | 0.9968 | 0.6111 | 0.6009 | 0.2508 |
| Cluster 2 | 2.20 X 10^-7^ | 0.6698 | 0.0785 | 0.1549 | 0.0023 |
| Cluster 3 | 0.8694 | 0.9968 | 0.0018 | 0.8600 | 0.0023 |
| Cluster 4 | 0.8694 | 0.9968 | 0.0069 | 0.7220 | 0.8603 |
| Cluster 5 | 0.8694 | 0.9968 | 0.0025 | 0.0089 | 0.1070 |
| **Six cluster solution** |  |  |  |  |  |
| Cluster 1 | 5.33 X 10^-10^ | 0.9968 | 0.6111 | 0.5408 | 0.3009 |
| Cluster 2 | 2.64 X 10^-7^ | 0.8037 | 0.0754 | 0.1526 | 0.0027 |
| Cluster 3 | 0.8982 | 0.9968 | 0.0011 | 0.8600 | 0.0027 |
| Cluster 4 | 0.8982 | 0.9968 | 0.0083 | 0.6931 | 0.8603 |
| Cluster 5 | 0.8982 | 0.9968 | 0.0011 | 0.1526 | 0.0702 |
| Cluster 6 | 0.5321 | 0.9968 | 0.0754 | 0.0012 | 0.3436 |
| **Seven cluster solution** |  |  |  |  |  |
| Cluster 1 | 6.22 X 10^-10^ | 0.9956 | 0.6111 | 0.5048 | 0.3511 |
| Cluster 2 | 3.08 X 10^-7^ | 0.9377 | 0.0879 | 0.1780 | 0.0032 |
| Cluster 3 | 0.8977 | 0.9956 | 9.54 X 10^-4^ | 0.8730 | 0.0032 |
| Cluster 4 | 0.5448 | 0.9956 | 9.54 X 10^-4^ | 0.8730 | 0.8603 |
| Cluster 5 | 0.9966 | 0.9956 | 0.1317 | 0.3220 | 0.8603 |
| Cluster 6 | 0.9966 | 0.9956 | 9.54 X 10^-4^ | 0.1780 | 0.0819 |
| Cluster 7 | 0.5448 | 0.9956 | 0.0879 | 0.0014 | 0.4009 |
| **Eight cluster solution** |  |  |  |  |  |
| Cluster 1 | 7.11 X 10^-10^ | 0.9956 | 0.6111 | 0.5769 | 0.3210 |
| Cluster 2 | 3.52 X 10^-7^ | 0.9956 | 0.0838 | 0.2035 | 0.0022 |
| Cluster 3 | 0.4550 | 0.9956 | 0.0011 | 0.8730 | 0.0022 |
| Cluster 4 | 0.9966 | 0.9956 | 0.0025 | 0.8730 | 0.0776 |
| Cluster 5 | 0.4981 | 0.9956 | 0.0011 | 0.8730 | 0.8603 |
| Cluster 6 | 0.9966 | 0.9956 | 0.1290 | 0.3680 | 0.8603 |
| Cluster 7 | 0.9966 | 0.9956 | 0.0011 | 0.2035 | 0.0776 |
| Cluster 8 | 0.4981 | 0.9956 | 0.0838 | 0.0016 | 0.3818 |
| **Nine cluster solution** |  |  |  |  |  |
| Cluster 1 | 8.00 X 10^-10^ | 0.9958 | 0.6111 | 0.5408 | 0.3009 |
| Cluster 2 | 3.96 X 10^-7^ | 0.9958 | 0.0808 | 0.1859 | 0.0025 |
| Cluster 3 | 0.5119 | 0.9958 | 9.99 X 10^-4^ | 0.8730 | 0.0025 |
| Cluster 4 | 0.9966 | 0.9958 | 0.0027 | 0.8730 | 0.1036 |
| Cluster 5 | 0.5604 | 0.9958 | 0.0012 | 0.8730 | 0.8603 |
| Cluster 6 | 0.9966 | 0.9958 | 0.1270 | 0.4140 | 0.8603 |
| Cluster 7 | 0.5668 | 0.9958 | 2.95 X 10^-4^ | 0.1073 | 0.1663 |
| Cluster 8 | 0.9966 | 0.9958 | 0.0027 | 0.4365 | 0.1036 |
| Cluster 9 | 0.5604 | 0.9958 | 0.0808 | 0.0018 | 0.3682 |
| **Ten cluster solution** |  |  |  |  |  |
| Cluster 1 | 8.89 X 10^-10^ | 0.9958 | 0.6790 | 0.5151 | 0.2866 |
| Cluster 2 | 3.25 X 10^-9^ | 0.7275 | 0.7892 | 0.3149 | 0.0064 |
| Cluster 3 | 0.4266 | 0.9958 | 7.40 X 10^-4^ | 0.8730 | 0.0055 |
| Cluster 4 | 0.9966 | 0.9958 | 0.0025 | 0.8730 | 0.0921 |
| Cluster 5 | 0.3037 | 0.9958 | 2.60 X 10^-4^ | 0.1996 | 0.0697 |
| Cluster 6 | 0.5189 | 0.9958 | 0.0010 | 0.8730 | 0.8603 |
| Cluster 7 | 0.9966 | 0.9958 | 0.1411 | 0.3680 | 0.8603 |
| Cluster 8 | 0.5398 | 0.9958 | 2.60 X 10^-4^ | 0.1193 | 0.1540 |
| Cluster 9 | 0.9966 | 0.9958 | 0.0025 | 0.4042 | 0.0921 |
| Cluster 10 | 0.5189 | 0.9958 | 0.0860 | 0.0020 | 0.3580 |

**Table S20.** Results of one-tail one-sample t-tests corrected for multiple comparisons (FDR) for the univariate task-related fMRI responses in the two to ten cluster solution for right hemisphere.

|  | Task 1 | Task 2 | Task 3 | Task 4 | Task 5 |
| --- | --- | --- | --- | --- | --- |
|  | (Taste vs Distilled water) | (High vs Low conc. sour) | (Grasp vs Reach) | (Vestibular stim. vs No stimulation) | (Lipsmack vs Scrambled stimuli) |
| **Two cluster solution** |  |  |  |  |  |
| Cluster 1 | 1.74 X 10^-12^ | 0.0190 | 0.1420 | 0.861 | 0.2365 |
| Cluster 2 | 0.9889 | 0.9913 | 3.99 X 10^-4^ | 0.0017 | 9.65 X 10^-4^ |
| **Three cluster solution** |  |  |  |  |  |
| Cluster 1 | 1.74 X 10^-12^ | 0.0190 | 0.1420 | 0.0861 | 0.2365 |
| Cluster 2 | 0.9845 | 0.9861 | 0.0135 | 0.0381 | 0.0073 |
| Cluster 3 | 0.9536 | 0.9475 | 1.63 X 10^-4^ | 0.0022 | 0.0072 |
| **Four cluster solution** |  |  |  |  |  |
| Cluster 1 | 1.74 X 10^-12^ | 0.0190 | 0.1420 | 0.0861 | 0.2365 |
| Cluster 2 | 0.9845 | 0.9861 | 0.0135 | 0.0381 | 0.0073 |
| Cluster 3 | 0.8770 | 0.2560 | 0.0043 | 0.0021 | 0.0045 |
| Cluster 4 | 0.9315 | 0.9764 | 7.78 X 10^-7^ | 0.0116 | 0.2430 |
| **Five cluster solution** |  |  |  |  |  |
| Cluster 1 | 1.74 X 10^-12^ | 0.0190 | 0.1420 | 0.0861 | 0.2365 |
| Cluster 2 | 0.9735 | 0.9684 | 0.0013 | 0.0706 | 0.0072 |
| Cluster 3 | 0.9698 | 0.9808 | 0.9046 | 0.0508 | 0.1740 |
| Cluster 4 | 0.8770 | 0.2560 | 7.78 X 10^-7^ | 0.0021 | 0.0045 |
| Cluster 5 | 0.9315 | 0.9764 | 0.0043 | 0.0116 | 0.2430 |
| **Six cluster solution** |  |  |  |  |  |
| Cluster 1 | 0.0018 | 0.7831 | 0.0458 | 0.0544 | 0.0011 |
| Cluster 2 | 1.53 X 10^-13^ | 0.0030 | 0.1904 | 0.1380 | 0.3782 |
| Cluster 3 | 0.9735 | 0.9684 | 0.0013 | 0.0706 | 0.0072 |
| Cluster 4 | 0.9698 | 0.9808 | 0.9046 | 0.0508 | 0.1740 |
| Cluster 5 | 0.8770 | 0.2560 | 7.78 X 10^-4^ | 0.0021 | 0.0045 |
| Cluster 6 | 0.9315 | 0.9764 | 0.0043 | 0.0116 | 0.2430 |
| **Seven cluster solution** |  |  |  |  |  |
| Cluster 1 | 0.0018 | 0.7821 | 0.0458 | 0.0544 | 0.0011 |
| Cluster 2 | 1.53 X 10^-13^ | 0.0030 | 0.1904 | 0.1380 | 0.3782 |
| Cluster 3 | 0.8679 | 0.9805 | 0.0499 | 0.0889 | 0.7243 |
| Cluster 4 | 0.9795 | 0.9309 | 2.34 X 10^-4^ | 0.1151 | 0.0010 |
| Cluster 5 | 0.8770 | 0.2560 | 7.78 X 10^-4^ | 0.0021 | 0.0045 |
| Cluster 6 | 0.9698 | 0.9808 | 0.9046 | 0.0508 | 0.1740 |
| Cluster 7 | 0.9315 | 0.9764 | 0.0043 | 0.0116 | 0.2430 |
| **Eight cluster solution** |  |  |  |  |  |
| Cluster 1 | 3.02 X 10^-4^ | 0.8947 | 0.8299 | 0.1313 | 0.0019 |
| Cluster 2 | 1.53 X 10^-13^ | 0.0030 | 0.1904 | 0.1380 | 0.3782 |
| Cluster 3 | 0.2003 | 0.5172 | 0.0055 | 0.0300 | 0.0043 |
| Cluster 4 | 0.8679 | 0.9805 | 0.0499 | 0.0889 | 0.7243 |
| Cluster 5 | 0.9795 | 0.9309 | 2.34 X 10^-4^ | 0.1151 | 0.0010 |
| Cluster 6 | 0.8770 | 0.2560 | 7.78 X 10^-4^ | 0.0021 | 0.0045 |
| Cluster 7 | 0.9698 | 0.9808 | 0.9046 | 0.0508 | 0.1740 |
| Cluster 8 | 0.9315 | 0.9764 | 0.0043 | 0.0116 | 0.2430 |
| **Nine cluster solution** |  |  |  |  |  |
| Cluster 1 | 3.02 X 10^-4^ | 0.8947 | 0.8299 | 0.1313 | 0.0019 |
| Cluster 2 | 2.22 X 10^-16^ | 0.0023 | 0.6140 | 0.1875 | 0.2531 |
| Cluster 3 | 0.2003 | 0.5172 | 0.0055 | 0.0300 | 0.0043 |
| Cluster 4 | 1.13 X 10^-4^ | 0.0201 | 0.0036 | 0.1292 | 0.8897 |
| Cluster 5 | 0.8679 | 0.9805 | 0.0499 | 0.0889 | 0.7243 |
| Cluster 6 | 0.9795 | 0.9309 | 2.34 X 10^-4^ | 0.1151 | 0.0010 |
| Cluster 7 | 0.8770 | 0.2560 | 7.78 X 10^-4^ | 0.0021 | 0.0045 |
| Cluster 8 | 0.9698 | 0.9808 | 0.9046 | 0.0508 | 0.1740 |
| Cluster 9 | 0.9315 | 0.9764 | 0.0043 | 0.0116 | 0.2430 |
| **Ten cluster solution** |  |  |  |  |  |
| Cluster 1 | 3.02 X 10^-4^ | 0.8947 | 0.8299 | 0.1313 | 0.0019 |
| Cluster 2 | 2.22 X 10^-16^ | 0.0023 | 0.6140 | 0.1875 | 0.2531 |
| Cluster 3 | 0.2003 | 0.5172 | 0.0055 | 0.0300 | 0.0043 |
| Cluster 4 | 1.13 X 10^-4^ | 0.0201 | 0.0036 | 0.1292 | 0.8897 |
| Cluster 5 | 0.8679 | 0.9805 | 0.0499 | 0.0889 | 0.7243 |
| Cluster 6 | 0.9795 | 0.9309 | 2.34 X 10^-4^ | 0.1151 | 0.0010 |
| Cluster 7 | 0.8770 | 0.2560 | 7.78 X 10^-4^ | 0.0021 | 0.0045 |
| Cluster 8 | 0.9698 | 0.9808 | 0.9046 | 0.0508 | 0.1740 |
| Cluster 9 | 0.8481 | 0.9657 | 0.0016 | 0.0065 | 0.2403 |
| Cluster 10 | 0.9267 | 0.9712 | 0.0645 | 0.1777 | 0.3696 |

**Table S21.** Results of one-tail one-sample t-tests for the univariate task-related fMRI responses in the two to ten cluster solution for left hemisphere.

|  | Task 1 | Task 2 | Task 3 | Task 4 | Task 5 |
| --- | --- | --- | --- | --- | --- |
|  | (Taste vs Distilled water) | (High vs Low conc. sour) | (Grasp vs Reach) | (Vestibular stim. vs No stimulation) | (Lipsmack vs Scrambled stimuli) |
| **Two cluster solution** |  |  |  |  |  |
| Cluster 1 | 1.07 X 10^-7^ | 0.4301 | 0.0130 | 0.3305 | 0.0040 |
| Cluster 2 | 0.8640 | 0.9956 | 9.82 X 10^-4^ | 0.0109 | 0.1642 |
| **Three cluster solution** |  |  |  |  |  |
| Cluster 1 | 2.23 X 10^-5^ | 0.6487 | 0.0090 | 0.6293 | 0.0118 |
| Cluster 2 | 8.80 X 10^-8^ | 0.1340 | 0.0628 | 0.0620 | 4.69 X 10^-4^ |
| Cluster 3 | 0.8640 | 0.9956 | 9.82 X 10^-4^ | 0.0109 | 0.1642 |
| **Four cluster solution** |  |  |  |  |  |
| Cluster 1 | 2.23 X 10^-5^ | 0.6487 | 0.0090 | 0.6293 | 0.0118 |
| Cluster 2 | 8.80 X 10^-8^ | 0.1340 | 0.0628 | 0.0620 | 4.69 X 10^-4^ |
| Cluster 3 | 0.8694 | 0.9968 | 0.0042 | 0.5776 | 0.8603 |
| Cluster 4 | 0.8041 | 0.9869 | 0.0010 | 0.0018 | 0.0642 |
| **Five cluster solution** |  |  |  |  |  |
| Cluster 1 | 8.89 X 10^-11^ | 0.4945 | 0.6111 | 0.3605 | 0.2006 |
| Cluster 2 | 8.80 X 10^-8^ | 0.1340 | 0.0628 | 0.0620 | 4.69 X 10^-4^ |
| Cluster 3 | 0.6412 | 0.7481 | 3.53 X 10^-4^ | 0.8600 | 9.12 X 10^-4^ |
| Cluster 4 | 0.8694 | 0.9968 | 0.0042 | 0.5776 | 0.8603 |
| Cluster 5 | 0.8041 | 0.9869 | 0.0010 | 0.0018 | 0.0642 |
| **Six cluster solution** |  |  |  |  |  |
| Cluster 1 | 8.89 X 10^-11^ | 0.4945 | 0.6111 | 0.3605 | 0.2006 |
| Cluster 2 | 8.80 X 10^-8^ | 0.1340 | 0.0628 | 0.0620 | 4.69 X 10^-4^ |
| Cluster 3 | 0.6412 | 0.7481 | 3.53 X 10^-4^ | 0.8600 | 9.12 X 10^-4^ |
| Cluster 4 | 0.8694 | 0.9968 | 0.0042 | 0.5776 | 0.8603 |
| Cluster 5 | 0.8982 | 0.9861 | 2.71 X 10^-4^ | 0.0763 | 0.0351 |
| Cluster 6 | 0.2660 | 0.9062 | 0.0602 | 1.95 X 10^-4^ | 0.2864 |
| **Seven cluster solution** |  |  |  |  |  |
| Cluster 1 | 8.89 X 10^-11^ | 0.4945 | 0.6111 | 0.3605 | 0.2006 |
| Cluster 2 | 8.80 X 10^-8^ | 0.1340 | 0.0628 | 0.0620 | 4.69 X 10^-4^ |
| Cluster 3 | 0.6412 | 0.7481 | 3.53 X 10^-4^ | 0.8600 | 9.12 X 10^-4^ |
| Cluster 4 | 0.3113 | 0.9927 | 4.09 X 10^-4^ | 0.8730 | 0.8603 |
| Cluster 5 | 0.9966 | 0.9956 | 0.1129 | 0.1840 | 0.8603 |
| Cluster 6 | 0.8982 | 0.9861 | 2.71 X 10^-4^ | 0.0763 | 0.0351 |
| Cluster 7 | 0.2660 | 0.9062 | 0.0602 | 1.95 X 10^-4^ | 0.2864 |
| **Eight cluster solution** |  |  |  |  |  |
| Cluster 1 | 8.89 X 10^-11^ | 0.4945 | 0.6111 | 0.3605 | 0.2006 |
| Cluster 2 | 8.80 X 10^-8^ | 0.1340 | 0.0628 | 0.0620 | 4.69 X 10^-4^ |
| Cluster 3 | 0.1706 | 0.4239 | 2.22 X 10^-4^ | 0.8029 | 5.53 X 10^-4^ |
| Cluster 4 | 0.9403 | 0.8900 | 0.0012 | 0.8638 | 0.0388 |
| Cluster 5 | 0.3113 | 0.9927 | 4.09 X 10^-4^ | 0.8730 | 0.8603 |
| Cluster 6 | 0.9966 | 0.9956 | 0.1129 | 0.1840 | 0.8603 |
| Cluster 7 | 0.8982 | 0.9861 | 2.71 X 10^-4^ | 0.0763 | 0.0351 |
| Cluster 8 | 0.2660 | 0.9062 | 0.0602 | 1.95 X 10^-4^ | 0.2864 |
| **Nine cluster solution** |  |  |  |  |  |
| Cluster 1 | 8.89 X 10^-11^ | 0.4945 | 0.6111 | 0.3605 | 0.2006 |
| Cluster 2 | 8.80 X 10^-8^ | 0.1340 | 0.0628 | 0.0620 | 4.69 X 10^-4^ |
| Cluster 3 | 0.1706 | 0.4239 | 2.22 X 10^-4^ | 0.8029 | 5.53 X 10^-4^ |
| Cluster 4 | 0.9403 | 0.8900 | 0.0012 | 0.8638 | 0.0388 |
| Cluster 5 | 0.3113 | 0.9927 | 4.09 X 10^-4^ | 0.8730 | 0.8603 |
| Cluster 6 | 0.9966 | 0.9956 | 0.1129 | 0.1840 | 0.8603 |
| Cluster 7 | 0.3779 | 0.7392 | 3.27 X 10^-5^ | 0.0239 | 0.0924 |
| Cluster 8 | 0.9661 | 0.9958 | 0.0015 | 0.2425 | 0.0460 |
| Cluster 9 | 0.2660 | 0.9062 | 0.0602 | 1.95 X 10^-4^ | 0.2864 |
| **Ten cluster solution** |  |  |  |  |  |
| Cluster 1 | 8.89 X 10^-11^ | 0.4945 | 0.6111 | 0.3605 | 0.2006 |
| Cluster 2 | 6.50 X 10^-10^ | 0.0727 | 0.7892 | 0.1260 | 0.0013 |
| Cluster 3 | 0.1706 | 0.4239 | 2.22 X 10^-4^ | 0.8029 | 5.53 X 10^-4^ |
| Cluster 4 | 0.9403 | 0.8900 | 0.0012 | 0.8638 | 0.0388 |
| Cluster 5 | 0.0911 | 0.3412 | 5.21 X 10^-5^ | 0.0599 | 0.0209 |
| Cluster 6 | 0.3113 | 0.9927 | 4.09 X 10^-4^ | 0.8730 | 0.8603 |
| Cluster 7 | 0.9966 | 0.9956 | 0.1129 | 0.1840 | 0.8603 |
| Cluster 8 | 0.3779 | 0.7392 | 3.27 X 10^-5^ | 0.0239 | 0.0924 |
| Cluster 9 | 0.9661 | 0.9958 | 0.0015 | 0.2425 | 0.0460 |
| Cluster 10 | 0.2660 | 0.9062 | 0.0602 | 1.95 X 10^-4^ | 0.2864 |

**Table S22.** Results of one-tail one-sample t-tests for the univariate task-related fMRI responses in the two to ten cluster solution for right hemisphere.
